# Emerging investigator series: automated single-nanoparticle quantification and classification: a holistic study of particles into and out of wastewater treatment plants in Switzerland†

**DOI:** 10.1039/d0en01066a

**Published:** 2021-03-23

**Authors:** Kamyar Mehrabi, Ralf Kaegi, Detlef Günther, Alexander Gundlach-Graham

**Affiliations:** Department of Chemistry and Applied Biosciences, ETH Zurich Switzerland; Department of Process Engineering, Eawag Dübendorf Switzerland; Department of Chemistry, Iowa State University Ames USA alexgg@iastate.edu

## Abstract

Single particle inductively coupled plasma time-of-flight mass spectrometry (sp-ICP-TOFMS), in combination with online microdroplet calibration, allows for the determination of particle number concentrations (PNCs) and the amount (*i.e.* mass) of ICP-MS-accessible elements in individual particles. Because sp-ICP-TOFMS analyses of environmental samples produce rich datasets composed of both single-metal nanoparticles (smNPs) and many types of multi-metal NPs (mmNPs), interpretation of these data is well suited to automated analysis schemes. Here, we present a new data analysis approach that includes: 1. automatic particle detection and elemental mass determinations based on online microdroplet calibration, 2. correction of false (randomly occurring) multi-metal associations caused by measurement of coincident but distinct NPs, and 3. unsupervised clustering analysis of mmNPs to identify unique classes of NPs based on their element compositions. To demonstrate the potential of our approach, we analyzed water samples collected from the influent and effluent of five wastewater treatment plants (WWTPs) across Switzerland. We determined elemental masses in individual NPs, as well as PNCs, to estimate the NP removal efficiencies of the individual WWTPs. From WWTP samples collected at two points in time, we found an average of 90% and 94% removal efficiencies of single-metal and multi-metal NPs, respectively. Between 5% to 27% of detected NPs were multi-metal; the most abundant particle types were those rich in Ce–La, Fe–Al, Ti–Zr, and Zn–Cu. Through hierarchical clustering, we identified NP classes conserved across all WWTPs, as well as particle types that are unique to one or a few WWTPs. These uniquely occurring particle types may represent point sources of anthropogenic NPs. We describe the utility of clustering analysis of mmNPs for identifying natural, geogenic NPs, and also for the discovery of new, potentially anthropogenic, NP targets.

## Abstract 2

High-throughput single-particle ICP-TOFMS analyses are used to quantify and classify diverse metal nanoparticle types from wastewater treatment plant samples.

## Graphical abstract

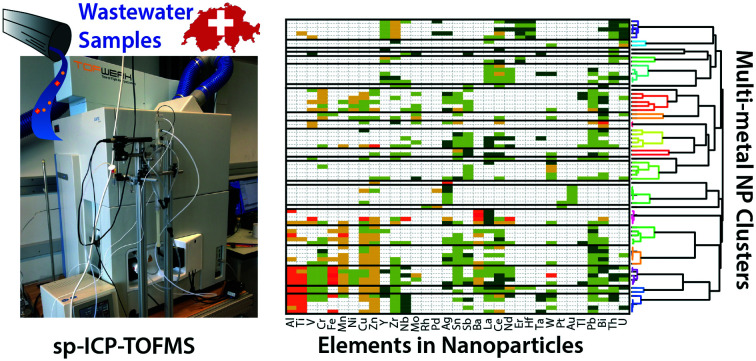

**Environmental significance**

The sp-ICP-TOFMS approach described here is a method for the determination metal- and metalloid-containing micro- and nano-particles (NPs) in environmental samples. The approach combines high-throughput measurement and quantification of individual particles with automated data analysis schemes to deliver non-targeted clustering and classification of NP types. Unique NP types are developed based on multi-element compositions measured in individual particles and the conservation of particle types between samples. Automated sp-ICP-TOFMS presents a means to detect and identify both expected and unexpected particle types within large particle backgrounds characteristic of *e.g.* surface waters. Key areas of impact include continued development of element-fingerprints to distinguish anthropogenic from natural NPs, high-throughput screening for monitoring nano-pollution, and discovery of novel particle targets in diverse environmental compartments.

## Introduction

The potential risk associated with the release of engineered nanoparticles (ENPs) into the environment impacts the development and proliferation of nanotechnologies. To assess this risk, researchers have developed nanomaterial-specific strategies^1^ that focus on exposure,^2,3^ hazards,^4,5^ and fate^6^ of ENPs in natural environments. Researchers estimate that up to 3 × 10^11^ g of ENPs go into landfills, soil, (waste)water, and air;^7^ though, these estimates may deviate by orders of magnitudes from the true values.^8^ A challenge in developing models to calculate mass flows of NPs is that they depend on manufacturers to disseminate accurate data about production and use of NPs, which is often not required. The most used NPs across all industries are carbon black, TiO_2_, Ag, SiO_2_, Fe(oxide), ZnO, Al_2_O_3_, carbon nanotubes, graphene, and nanoclays;^3,9,10^ however, there are many more types of ENPs. There are also many incidental NPs (INPs) that are produced as a byproduct of human activities that remain unknown and/or have not been studied sufficiently to accurately predict their contribution to system-wide models of NP fate and transport.^6^ Hence, we need appropriate analytical methods to monitor NP pollution in and into the environment—such as through pathways like waste water treatment plants (WWTPs)^11^ and landfills^12^—and also methods to search for and identify possible anthropogenic NPs.

There are two basic approaches to measure NPs in the environment: either through bulk measurements or particle-by-particle approaches. For bulk measurements, size fractionation followed by analytical measurement is often used to determine abundance of particulates and dissolved species in a sample; this fractionation may be off-line such as with serial filtration steps or may be accomplished online with separation procedures such as size exclusion chromatography or field flow fractionation.^12,13^ Analytical measurements of fractions can be accomplished with either non-specific detection such as with light scattering, or element-specific detection such as with ICP-OES or -MS. Bulk methods can provide an overall picture of the possible NP release, but generally can't be used to detect low particle number concentrations (PNCs), don't provide direct information about individual NPs, and also may produce biased results based on selectivity of the filter.^14^ Particle-by-particle measurements include approaches such as scanning and transmission electron microscopies^15^ and single-particle ICP-MS (sp-ICP-MS).^16–18^ Electron microscopy techniques, with the help of an energy dispersive spectrometer, can provide information on shape, size, crystal structure, and elemental composition of individual NPs. However, these approaches are low throughput, which limits—even the most sophisticated automated setups—to PNC detection limits of ∼10^6^ particles mL^−1^, and the detection of target particle types/compositions against an overwhelming number of ‘background’ NPs, as is observed in natural samples, is especially challenging.^19^

Single-particle-ICP-MS enables direct measurement of analyte element mass in individual NPs and offers high throughput detection, with PNC detection limits down to ∼10^2^ particles mL^−1^. However, sp-ICP-MS alone provides no information on particle size or morphology. In combination with *m*/*z*-dispersive mass spectrometry with multi-channel detection, such as time-of-flight mass spectrometry (TOFMS), sp-ICP-MS can be used to quantitatively determine the amounts of several elements and isotopes in single NPs. In ICP-TOFMS, the complete elemental mass spectrum (from Li to U) is recorded continuously at time resolutions down to 1 ms. At this time resolution, ICP-TOFMS can be used to record NP-derived signals, which are short transients typically between 300–500 μs in duration.^20^ Through multi-element and multi-isotope detection, sp-ICP-MS provides a more comprehensive analysis of the composition of individual NPs, which is ideal for classifying and sorting NPs, especially those with partially overlapping element compositions. Still, with ICP-MS, some elements commonly present in NPs, such as carbon, nitrogen, oxygen, sulfur, and fluorine, are not readily detectable at the single-particle level.^21^ Throughout this manuscript, we use the terms “single-metal” and “multi-metal” NPs (smNP and mmNP) to refer to particles measured with just one and with two or more ICP-TOFMS-detectable elements, respectively.

Being able to distinguish between naturally occurring NPs (NNPs) and engineered NPs (ENPs) has been the topic of many studies.^10,22–29^ Although there is ambiguity on the best method of investigation of these particles types, the most prominent NP characteristics used to categorize a NP as either engineered or natural include composition, crystal structure, particle morphology, and shape.^30,31^ An advantage of sp-ICP-TOFMS is that one can simultaneously measure most of the metal and metalloid elements in single run, which means that non-targeted analysis can be used to detect variable and unique elemental fingerprints of different particle types. Due to manufacturing processes, it is assumed that ENPs will have fairly pure and controlled elemental fingerprints, which will differ from those of NNPs.^16,25^ For example, a previous study on Ce-containing NPs demonstrated that prominent Ce-containing NNPs also contain La, Pr, Nd, and Th, whereas in CeO_2_ ENPs only Ce was detected.^25^ Researchers have also focused on the measurement of Ti-containing NPs and the discrimination of Ti-containing NNPs and TiO_2_ ENPs.^14,22,26–28,32^ In one such study, researchers demonstrated that Ti-containing NNPs are associated with Mn, Fe, V, and Pb;^14^ in another study, researchers suggested that Nb is an indicator of Ti-containing NNPs and such NPs could also contains traces of Ta, W, and other elements.^27^ In fact, both of these elemental associations are consistent with geochemical knowledge: titanium iron oxide (*e.g.* ilmenite, FeTiO_3_) often has Mn and V impurities, while naturally occurring titanium oxide (*e.g.* rutile, TiO_2_) has common impurities of Nb, Ta, and W, among others.^33,34^ The uncertainty and discrepancies reported in elemental fingerprints of natural and anthropogenic Ti-containing NPs illustrate a challenge in multi-metal sp-ICP-TOFMS: the large amount of data generated requires an automated and robust data processing strategy. Human-based evaluation of elemental associations through visual inspection of data and user-based pattern recognition is extremely time consuming and is prone to user error and bias.^32^ For example, a user might limit the analyte element list to simplify multi-metal patterns in the data and so miss unanticipated multi-metal associations. Similarly, low-abundance multi-metal associations are more likely to be overlooked in non-automated data analysis schemes.

Here, we present a high throughput data acquisition and data evaluation approach for non-targeted analysis of NPs measured *via* sp-ICP-TOFMS. We discuss the critical steps required to process raw ICP-TOFMS data into quantified information on NPs, and then we apply these techniques to extract NP information from sp-ICP-TOFMS measurements of influent and effluent from five waste water treatment plants (WWTPs) across Switzerland. Our method delivers high-throughput, *in situ* characterization of NP populations in order to provide holistic (*e.g.* comprehensive) datasets on inorganic NPs reaching and passing WWTPs. Through the analysis of mmNP compositions, we report a new means—*via* hierarchical clustering—to discover both conserved and unique mmNP types across the wastewater samples. This mmNP clustering approach provides new insights into the origins of various particle types present in the wastewater samples. With sp-ICP-TOFMS, we determine element mass(es) per particle. Here, we do not convert measured mass to particle diameters; however, if a particle stoichiometry and density were known or assumed, then element mass could be converted into an equivalent spherical particle diameter. Throughout this manuscript, we use the term “nanoparticle” (NP) to discuss all measured particle signals because we estimate that the majority of detected particles have diameters ranging from a few tens to hundreds of nanometers. Measured “NPs” by sp-ICP-TOFMS would be more completely described as a combination micro- and nano-particles, depending on the working definition used.

## Materials and methods

### Sample selection and preparation

Based on a previous screening study of element concentrations in WWTPs in Switzerland by Vriens *et al.*,^35^ complemented by detailed investigations of Ce-containing NPs in selected sewage sludge samples,^23^ we selected five WWTPs for this study (Table 1). A map of the locations of these WWTPs is provided as Fig. S1 in the ESI.† Further details on the locations, catchment sizes, and average major-, minor-, and trace-element concentrations in sludge and effluent can be found in the report by Vriens *et al.*^35^ These WWTPs (W1–W5) have been previously reported to have high concentrations of Ce and La in sludge samples, and results from synchrotron X-ray absorption spectroscopy studies have suggested that these WWTPs had high industrial Ce-NP concentrations based on the oxidation state of Ce and the ratio of Ce to other rare-earth elements (REEs).^23^

**Table 1:** Data of the WWTPs where influent and effluent samples were collected (taken from Vriens *et al.*)

WWTP (effluent, influent)	Location	Altitude (m a.s.l.)	Average daily waste water inflow (m^3^ d^−1^)	Average sludge production (kg d^−1^)	Connected population equivalents	Treatment type
**W1 (E1,I1)**	Yverdon	431	10 000	840	10 000	Mechanical-biological with Phosphorus elimination
**W2 (E2,I2)**	St. Gallen	580	21 850	12 900	37 057	Mechanical-biological with Phosphorus elimination
**W3 (E3,I3)**	SG-Hofen	596	27 145	20 000	52 006	Mechanical-biological with Phosphorus elimination
**W4 (E4,I4)**	Buchs	445	850	2100	24 000	Mechanical-biological with Phosphorus elimination and nitrification
**W5 (E5,I5)**	ProRheno (Basel)	250	90 787	36 255	246 042	Mechanical-biological with Phosphorus elimination

From each WWTP, we collected influent (I) samples after primary clarification and effluent (E) samples in 500 mL glass media bottles and measured the samples by sp-ICP-TOFMS on the day of collection. Prior to sp-ICP-TOFMS analysis, we sonicated 2 mL aliquots of samples in 2 mL polypropylene centrifuge tubes for a total sonication time of 15 seconds at 100% sonication power (200 W, UP200St VialTweeter Sonotrode, Hielscher Ultrasound Technology, Germany). The sonicated samples were allowed to settle for 10 minutes and then the top 1 mL of the sonicated samples was diluted into 8.9 mL of DI water and spiked with a 100 μL of a nominally 100 ng mL^−1^ solution of Cs for a final Cs concentration of 1 ng mL^−1^ in each sample. All samples were diluted 10 times; in addition, due to the high particulate contents, we diluted samples from I1 and I2 from the Nov. sampling by 100 times. The Cs standard was made in DI water from standard stock solution of 1000 μg mL^−1^ (Inorganic Ventures, Christiansburg, VA, USA). All dilutions were made gravimetrically. Microdroplet solutions were prepared from 1000 μg mL^−1^ single-element standard solutions (Inorganic Ventures, USA) in trace-grade 3% HCl (TraceSelect, Fluka Analytical, Switzerland) and in-house sub-boiled 1% HNO_3_ (DuoPUR sub-boiling distillation system, Milestone GmbH, Germany).

### Sample introduction and MS measurement

Measurements were performed on an icpTOF-2R (TOFWERK AG, Thun, Switzerland) instrument, which has a native mass resolving power (*m*/Δ*m* at FWHM) of ∼6000 and an extraction frequency of 21.739 kHz. Element-mass and PNC determinations were performed according to our previously published online microdroplet calibration method;^36^ additional details of experiment conditions are provided in Table S1.† Briefly, we used a dual sample introduction setup, which was composed of nebulizer/spray chamber for wastewater sample introduction and a falling tube and microdroplet generator (50 μm diameter Autodrop Pipette, AD-KH-501-L6, Microdrop Technologies GmbH, Germany) for introduction of monodisperse microdroplets. The elements in the microdroplet calibration solution and their concentrations are provided in Table S2.† Droplets were introduced into a vertical falling tube filled with a helium/argon gas^37^ mixture and then introduced into the plasma concomitant with sample aerosols from the conventional pneumatic nebulizer/cyclonic spray chamber. A known amount of a plasma-uptake standard (*i.e.* cesium, Cs) was spiked into the WWTP samples and the microdroplet calibration solution. Through detection of the nebulized and microdroplet-contained Cs, we determined sample plasma uptake rate (*q*_plasma_) for every sample, which allowed us to directly calculate the PNCs. Each wastewater sample was measured for a total time of 450 seconds in three 150 second replicates. Plasma uptake rates for all analyses are reported in an excel sheet included in the ESI.†

### Data processing

Following sp-ICP-TOFMS measurements, an automated data analysis process was followed to detect, quantify, and cluster smNPs and mmNPs. We list the basic steps of our data analysis procedure below. A detailed explanation of each of these data processing steps is beyond the scope of this report—in part because many aspects of the analysis strategy are reported elsewhere.^36,38–42^ For aspects of this process that are new to the current study, we provide a more detailed discussion below. In addition, our “NanoFinder” software is provided under an open-source license (http://doi.org/10.5905/ethz-1007-363) and is hosted at https://github.com/ethz-tema/TEMAsingleParticle.

1. Select elements and isotopes of interest, generate time traces of selected elements.

2. Determine critical value (*L*_C_) expressions based on compound-Poisson modelling.^38,41^

3. Determine background (dissolved signal) count rates (*λ*_bkgd_) for all elements.

4. Background subtract all time traces.

5. Find microdroplet signals, determine mass sensitivities for each element i (*S*_drop,i_) and *q*_plasma_.^36,39,40^

6. Correct all time traces for split events.^42^

7. Find NP signals above the single-particle critical value (*L*_c,sp_).

8. Correct data set for particle-coincidence to remove spurious mmNP signals caused by concurrent measurement of two or more discrete particles with unique element fingerprints.

9. Quantify elemental masses from detectable individual NP signals.

10. Perform hierarchical clustering analysis of mmNP signals; discovery of conserved and non-conserved mmNP types.

11. Quantify detectable PNCs of both smNPs and mmNPs.

12. Report smNP and mmNP data.

Automated data analysis is critical for high-throughput sp-ICP-TOFMS because of the amount of data obtained. For example, let us consider the analysis of just one of our datasets: one replicate of sample I5. From this ICP-TOFMS dataset, we selected 33 elements of interest, which requires the determination of 33 independent sample-specific background count rates (*λ*_bkgd_) and the associated critical values (*L*_c,sp_) to threshold the data for particle signals. In each time trace, we extracted microdroplet signals and determined matrix-matched absolute mass sensitivities (*S*_drop,i_) and plasma uptake rates (*q*_plasma_). The single-particle region of each time trace contains 75 000 data points, *i.e.* 2.475 × 10^6^ data points for all elements combined. For each of these time traces, we background-subtracted the signals, parsed through the data to correct for split events, and collected NP signals above *L*_c,sp_. For sample I5, we found 45 008 particle signals. We identified mmNPs based on concurrent particle signals for two or more elements, and then corrected these events to account for predicted particle coincidences. For I5, we found 8215 mmNPs, of which 6085 are true mmNPs. After identifying the mmNPs, we compiled the element combinations of these particles: of the 6085 mmNPs, there were around 820 unique mmNP element combinations. This list of mmNP element combinations is cumbersome, and was, thus, reduced through hierarchical clustering. These data processing steps were repeated for every replicate and sample analyzed. Clearly, is not possible to sift through sp-ICP-TOFMS data by hand. For data from the 10 samples (I1–5 and E1–5) presented here, we made 30 sp-ICP-TOFMS measurements, *i.e.* we processed data spread across 990 unique time traces and composed of a total of 2.45 billion data points. With automatic and robust sp-ICP-TOFMS quantification, we were able to process large data files and reduce these data to a reportable format that can be used to help interrogate the data with regard to scientific questions such as understanding of particle inputs, compositions, transport properties, *etc.*

### Element selection and critical value determination

From sp-ICP-TOFMS data, we found 33 elements that occurred frequently as particle-events; a list of these elements and the isotopes selected is presented in Table S2.† As previously mentioned, not all elements present in NPs are detectable by sp-ICP-TOFMS. Specifically, with our ICP-TOFMS setup, some elements that are predicted to be associated with NNPs and ENPs (*e.g.* O, F, Si, P, and S) could not be measured at the single-particle level. The absence of data on these elements in sp-ICP-TOFMS data does not mean that these elements are not present in measured NPs, but rather that they just weren't detected.

For each element, we calculated single-particle critical values (*L*_c,sp_) based on a compound Poisson distribution.^38^ As previously reported, the critical value for a given false-positive (alpha) rate can be determined as a linear relationship with the *λ*_bkgd_, as shown in the generalized eqn 1.

1 *L*_C,sp(@α′)_ = *m*_(@α′)_(*λ*_bkgd_)^1/2^ + *b*_(@α′)_

The critical value expression is dependent on the false-positive rate (*α*) modelled for the compound Poisson distribution, and so we determined the slope (*m*_(@*α*′)_) and intercept (*b*_(@*α*′)_) for a set of false-positive rates, as presented in presented in Table S3.† The critical value expression is estimated based on the response function of the TOFMS detection system, and so one expression may be used for operation of the TOFMS instrument with constant settings over a day. For the analysis of each sp-ICP-TOFMS time trace, we set element-specific *L*_c,sp,i_ values that feature dynamically adjusted false positive rates (*α* values) in order maintain the relationship *f*_p_ ≥ 40 × *α*, where *f*_p_ is the frequency of particle-events measured. For example, if, for some element, 75 particle-events were detected out of 75 000 data points, then *f*_p_ would be 0.001 and the *α* value would be set to a maximum of 0.000025. At this false positive rate, we would predict just 1.875 false detection events and so expected error in the 75 detected NPs would be ∼2.5%. Use of dynamic element- and sample-specific critical values allows us to balance control of false-positive errors, while also finding more low-intensity particle signals. During initial data evaluation, we examined data with more conservative f_p_/*α* values of 100 and 400, *i.e.* 1% and 0.25% particle-events to predicted false positives, but did not observe substantial differences in found NP classes. Count-based *L*_c,sp,i_ values for all measured elements in all samples are provided in an Excel spreadsheet in the ESI.† In Fig. S5,† we provide the critical values in terms of mass for each element in each WWTP sample. Additionally, in Fig. S6,† we provide the average equivalent-mass spherical diameters of elements in their major oxide forms at their respective critical values. For most elements, these diameter critical values (akin to detection limits) are below 50 nm; notable exceptions include particulate Al, Ti, and Fe.

### Quantification of element mass in individual NPs

Determination of the mass of selected elements in individual NPs was accomplished according to eqn 2, in which *I*_p,i_ is the background-subtracted intensity of element i in an individual NP, *S*_drop,i_ is the background-subtracted sensitivity of element i from the microdroplet standards, and *m*_p,i_ is the mass of element i in the particle.2
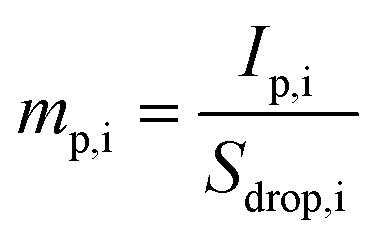
For online microdroplet calibration, our microdroplets were composed of a solution with known concentrations of Al, Ti, Fe, Cu, Zn, Ag, Sn, Cs, Ce, Pt, Au, Pb, and U. These elements were selected for our calibration solution because they cover a broad mass range and because they include many elements of interest initially assumed to be present in form of NPs in the WWTP samples (*e.g.* Al, Ti, Fe, Zn, and Ce). To determine the mass(es) of elements found as NPs, but not present in the microdroplet calibration solution, we estimated missing element sensitivities based on linear interpolation of measured microdroplet-based sensitivities *versus* element mass (Fig. S2†). For this sensitivity calibration, we divided the instrument responses into three sections according to mass-to-charge (*m*/*z*) ranges in order to minimize broad-range non-linear effects of instrument mass bias. These three sections are low (*m*/*z* <63 Th), mid (63 Th < *m*/*z* <133 Th) and high (*m*/*z* >133 Th). We also excluded elements present in microdroplets that have high first ionization energy (*i.e.* Zn: 9.39 eV, Pt: 8.96 eV, Au: 9.23 eV) from the list of known responses for optimum response curve. We estimate that elements with interpolated sensitivities can be quantified within ±30% of true element mass, and so are considered semi-quantitative. Importantly, for single-particle analysis, the counts measured for elements in individual NPs are low, and the error based on counting statistics alone is often >10% (*i.e.* for element signals with less than 100 counts). Semi-quantitative analysis of individual NPs offers a way to normalize NP data in order to compare particle signals across samples and measurement periods, and so improves investigations of trends in sp-ICP-TOFMS data and classification strategies.

### Hetero-particle coincidence correction (hpCC)

In sp-ICP-TOFMS, a mmNP is found if two or more elements produce single-particle signals (*i.e.* signal intensities above the *L*_C,sp,i_) that are measured within the same time interval. However, such a mmNP event could arise from one of two scenarios: either it is a true mmNP, *i.e.* the multiple elements detected represent a true multi-element composition of the measured particle, or it is a false mmNP that is caused by the detection of two (or more) distinct NPs that happen to enter the plasma at the same time (within the time resolution of the measurement). We term this false mmNP case a particle-coincidence event. Importantly, particle coincidence could be one of two types. In type 1, a particle event is coincident with its own kind (*i.e.* identical element fingerprint). In type 2, two particle events with different element fingerprints are coincident with each other. Unlike type 1 particle coincidence, which can be only overcome through dilution of the sample or, to some extent, through higher MS time resolution,^43^ type 2 particle coincidence (*i.e.* hetero-particle coincidence) can be estimated and then eliminated based on a hetero-particle coincidence correction (hpCC).^36^

If we assume that particle coincidence events are random, then the probability of such events can be calculated following eqn 3, in which ***P***_**A**_ and ***P***_**B**_ are the probabilities of measuring particles **A** and **B** independently.^36^ In our data set, we compute the probabilities of different hetero-particle combinations: including smNPs overlapping to form false binary mmNPs, and also the likelihood of true mmNPs coinciding with smNPs and other mmNPs. To accomplish this task, we have developed a hpCC algorithm to predict the numbers of expected false mmNPs for each mmNP signature, as well as to rank the likelihood of whether individual mmNPs are true or false based on the similarities of element-abundance ratios between specific events and the composite mmNP group. A more complete discussion of our hpCC algorithm is provided in the ESI† and in Fig. S3. After hpCC, the numbers and recorded compositions of both smNPs and mmNPs more accurately describe the true smNP and mmNP populations. We use hetero-particle coincidence corrected NP data in all subsequent analysis steps.

3 *P*_A∩B_ = *P*_A_ × *P*_B_

### Hierarchical clustering for classification of mmNP types

We only clustered the multi-metal particles (mmNPs). For smNPs, we obtain mass distributions and PNCs; however, this information is not sufficient to develop meaningful clusters of the smNPs. Therefore, smNPs can be thought of as each representing their own particle cluster. If we had additional information of smNPs, such as shape or crystal structure, we could consider developing clustering approaches to identify the most similar smNP families.

For clustering analysis, we processed mmNP datasets through two-stage hierarchical clustering (HC) using a standard hierarchical clustering library in MATLAB (verR2020b Mathworks, MA, USA). A schematic of this HC process is provided as Fig. S4.† In the first step of HC, we listed the quantified elemental masses present in each mmNP to generate fundamental clusters that best account for variance in mmNP composition and frequency in each sample. Replicates of each sample were pooled for clustering analysis. For intra-sample clustering, we used the correlation distance and averaging method. After calculating the average correlation distance between all the particle-events, we generated an agglomerative hierarchical cluster tree, in which each particle is connected to the cluster tree by a single linkage and linkages are merged together to minimize average distance between all mmNPs. “Distance” represents the similarity of mmNPs to one another; the smaller the distance between two mmNPs the more similar they are. In agglomerative clustering, a cutoff threshold is required to divide the dendrogram into recognized clusters; we use a distance cutoff of 0.85 to identify major clusters in each wastewater sample. For influent samples, the intra-sample HC analyses resulted in 11–17 major clusters. In order to compare the similarities between the mmNP clusters developed for the different WWTPs, we performed a second inter-sample hierarchical clustering analysis. In this second analysis, we performed HC analysis on representative mmNP proxies that were pooled from each of the initial intra-sample clusters. The mmNP proxies were composed of elements that occur in more than 10% of the particles that are part of given intra-sample cluster. The amplitude of each element in a mmNP proxy is the median of the ratio of element mass to the mass of the normalizing element in individual particles. The normalizing element is cluster specific and is the element with the highest occurrence frequency in a particular cluster. For inter-sample clustering of the mmNP proxies, we again used the correlation distance metric with average unweighted linkages, but used a lower cluster cutoff of 0.5 to define the major clusters. Subgroups of the major intra-sample clusters, which break up the major clusters into contributions from different WWTPs, are apparent below correlation distances of 0.5 on the resultant dendrogram, which we will discuss in more detail below.

## Results and discussion

### Single-metal *vs.* multi-metal nanoparticles

**Fig. 1:**
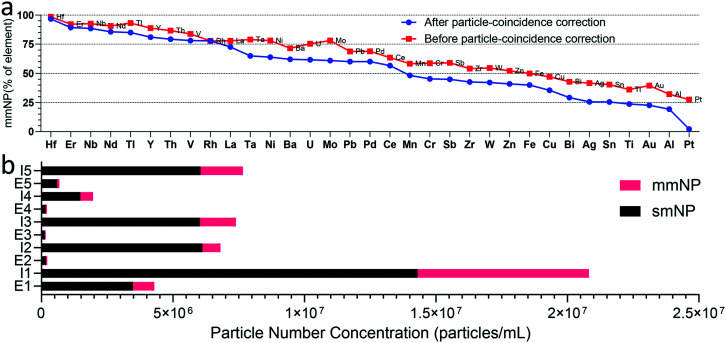
Overview of detected NPs across different wastewater samples. a) Percentage of mmNPs out of the total detected NPs for each element before and after particle-coincidence correction. b) Comparison of total mmNP and smNP PNCs in influent and effluent of WWTPs. Each mmNP is composed of at least two elements; however, it still counts as one particle.

Based on Poisson likelihood, there is a possibility that more than one particle will enter the plasma at the same time. If these multi-particle events are not identified, then they will be falsely registered as multi-metal events. To minimize the effect of these occurrences, we use a hpCC approach, as described in the methods section and ESI.† In Fig. 1a, we provide an overview of all the NPs detected in the influent wastewater samples and show the multi-metal portion of each detected element before and after hpCC. Up to 20% of the multi-metal events were predicted to be related to coincident particle events rather than true mmNP events. To reduce coincident-particle events, the samples can be diluted; however, without extending measurement time, this would limit the number concentration detection limits for low-abundance NP types. Increasing measurement time decreases sample throughput and could increase potential for sensitivity drift, which complicates particle-event thresholding. Through hpCC, we are developing a methodology to perform multi-particle ICP-TOFMS. This approach aims to expand the number concentration dynamic range available for all NP types and, in so doing, improve the throughput of measurements. Because hpCC cannot be used to correct for coincidence between particles with identical composition, we still must limit single-element particle frequencies to a maximum rate of ∼5–10% particle events in a single analysis; we limit these rates through dilution.^43^

In Fig. 1a, elements are presented from the left to the right according to the fraction of their NPs recorded as mmNPs. Even after hpCC, many elements have high mmNP fractions, meaning that these elements are most often present as mmNPs in our samples. All elements to the left of Ba have mmNP fractions >75%. The fact that these elements are often associated with mmNPs is in agreement with geochemical knowledge: REEs such as Er and Nd, mostly appear in combination with other REEs, as we find in naturally occurring NP signatures.^44^ On the right side of Fig. 1a, we provide the elements that are least likely to exist as mmNPs: these elements mostly occur as smNPs. For example, Pt and Au occur as mmNPs less than 25% of the time; these elements are noble metals and are often produced as single element ENPs.^45^ Ti and Al, which are present at two of the highest PNCs in these samples, also are measured as mmNPs less than 30% of the time. The fact that Al and Ti are highly present as smNPs could indicate influxes of engineered Al- and Ti-containing NPs, or it could be that associated elements with these NPs are below measurement detection limits. Between the extremes of abundant mmNP or smNP fractions, there are some elements—such as Ce and Zn—that have moderate mmNP fractions (between 40–60% mmNPs). These elements are known to exist as both smNPs and mmNPs;^10,25^ an even distribution of smNP and mmNP types could indicate an equal contribution (on a particle-number basis) of natural and anthropogenic NPs in the wastewater samples. Or again, this could be an artifact caused by associated elements at masses below the critical value in some of the registered smNPs.

The mass distributions of all elements in smNPs and in mmNP in both the influent and effluent samples are presented in Fig. S5.† As shown, the median mass of each element tends to be higher in mmNPs compared to that in smNPs. This can, to some extent, be explained by measurement bias of element fingerprints: in small NPs, a major-constituent element could be present at a large enough mass to be detected, but minor-constituent elements could have mass below their critical values. In this case, a true mmNP would be detected as a smNP. For larger NPs, the masses of major and minor-constituent elements will increase proportionally, and so it is more likely minor constituents will be measured, *i.e.* a mmNP will be recorded. Because the large NPs are more likely to have measurable amounts of minor-elements, the element mass distribution in mmNPs will tend to be higher. Importantly, for NPs from which at least two elements from the element fingerprint are recorded, variability in measured elements according to particle size is less critical because, as we discuss below, clustering analysis enables mmNPs with variable associated elements to still cluster into accurate NP classes.

### WWTPs in terms of nanoparticle removal

**Fig. 2:**
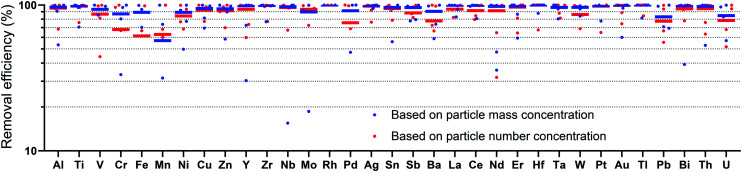
Removal efficiencies of different elements across the five WWTPs sampled in Nov. 2019. The average removal efficiency is indicated by a solid dash for each element. The large scatter in the data toward lower removal efficiencies is due to low numbers of particles measured for these elements.

Mass flow models suggest that a large fraction of ENPs are released to sewer systems,^3^ and will eventually be collected by WWTPs. Spiking experiments using different types of ENPs revealed their efficient removal (>95%) during the activated sludge process resulting in an accumulation of the ENPs in the sewage sludge. Here, we report results from multi-element measurement of NPs from the influent (I) after primary clarification and effluent (E) of full-scale WWTPs, which allows an estimation of the removal of NPs during the wastewater treatment. Because our influent and effluent samplings were collected at the same time, they do not reflect measurements of the same water parcel followed through the WWTPs. Possible temporal variabilities of the NP concentrations in the influent especially related to industrial discharges may, therefore, lead to biased removal efficiency determinations. To partially account for this sampling limitation, we verified our results with sp-ICP-TOFMS analysis of two samples from each of the WWTPs collected five months apart, in May and Nov. 2019. Data from the May sampling is provided in the ESI.† In Fig. 1b, we present an overview of the total smNP and mmNP signals found in the influent and effluent of each WWTP. In Fig. 2, we plot a comparison of the average removal efficiencies calculated based on particle number *versus* that based on mass concentrations of particles. The data in Fig. 2 are not distinguished by particle type (*i.e.* as smNPs or mmNPs), but rather only distinguished based on element composition. We find that NP removal efficiencies based on particle mass concentrations are higher than those based on number concentration, which suggests that larger NPs are, generally, retained to a greater extent in the WWTPs than smaller NPs. For most elements, removal efficiencies are between 75 and 100%. The lower removal efficiencies based on particle number highlights an advantage of single-particle analysis over bulk metals determination: with bulk analysis, low-mass particles have a minimal impact on determined removal efficiencies, whereas particle-number-based results better equalize contribution of small particles. Comprehensive element-specific single-particle analysis represents a new characterization possibility for analyses of NPs in WWTPs or other environmental compartments, and so the utility of this analytical approach will continue to expand.

**Fig. 3:**
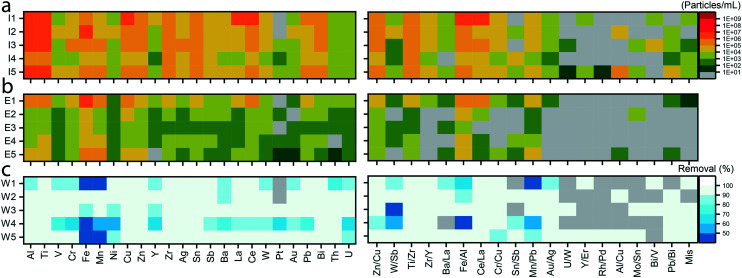
smNPs and mmNPs detection across different WWTPs. Heat maps show the PNCs in the influent (a) and effluent (b), and the PNC-based removal efficiency of each particle type in percentage (c). mmNP clusters are labeled according to the two most abundant elements measured in each particle cluster. Classification of the mmNPs is obtained *via* hierarchical clustering analysis as described in text (*cf.*Fig. 4).

In Fig. 3, we present PNCs for several smNPs and clusters of mmNPs measured in the influent and effluent of the five WWTPs from samples collected in Nov. 2019. (Analogous data from samples collected in May 2019 are reported in Fig. S7†). While we find many of the same particle clusters in the influent and effluent, there are dramatically fewer NPs in the effluent. This more detailed view of removal efficiency by particle type demonstrates that, in our analyses, NPs composed primarily of Al, Ti, Fe, Cu, Zn, and Ce are the dominant particle types in both influent and effluent, but are efficiently removed in most cases. (It is likely that silicate (SiO_2_) NPs, which we could not measure at the single-particle level due to low sensitivity at *m*/*z* 28 with the instrument settings used, would also be a dominant NNP species). Interestingly, some particle types seem to be less retained in WWTPs than others. For example, Fe-containing smNPs are less well removed than other particle types in three WWTPs from the Nov. sampling and in all the WWTPs from the May sampling. The apparent low removal efficiency of Fe NPs in terms of particle number could be due to the addition iron in the wastewater treatment process to retain phosphorus;^46^ some of the created ‘Fe–P’ colloids may pass the wastewater treatment. Through the characterization of mmNPs, we also find that most NP types are removed efficiently from the wastewater stream. Overall, the most abundant mmNP types include Ti–Zr, Zn–Cu, Al–Fe, and Ce–La. Conservation of NP type and abundance across all WWTPs suggests that the NPs are naturally occurring and ubiquitous. On the other hand, we find some NP types such as of Rh–Pd or Bi–V in just one or two WWTPs, which suggests that these NP types originate from specific (anthropogenic) point sources.

**Fig. 4:**
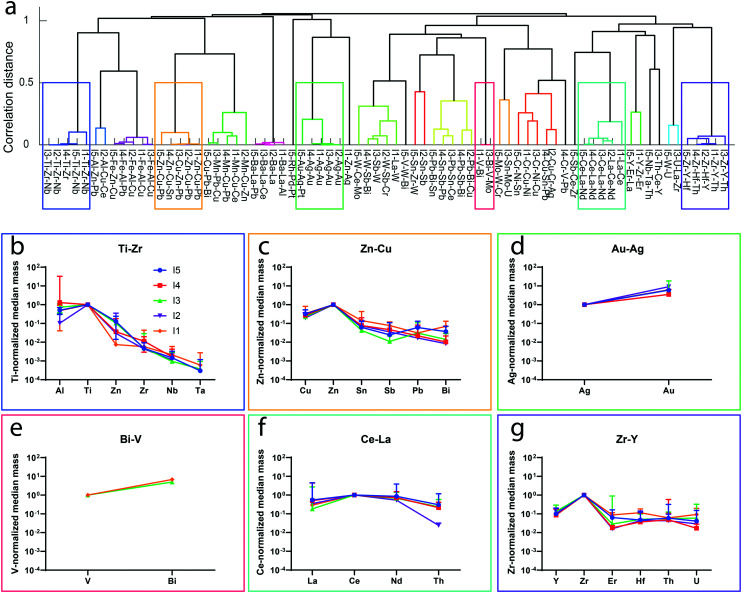
a) Dendrogram of inter-sample hierarchical clustering analysis of I1–I5 samples collected in Nov. 2019. In this cluster tree, we identify 23 major mmNP types with correlation distances above 0.5. Each unique mmNP type is plotted in a different color. Six of the unique mmNP clusters are highlighted in colored rectangles. For these mmNP clusters, the representative mmNP proxies with elements present at an occurrence rate limit of 1% are plotted in b–g. Note that not all mmNPs types are found in all WWTP influent samples. While the Ti–Zr, Zn–Cu, Au–Ag, Ce–La, and Zr–Y mmNP clusters are found in all influent samples, the Bi–V-type mmNPs are only found in I1 and I3. Error bars in b–g are standard deviations of normalized masses.

### Automated clustering to discover multi-metal nanoparticle types

The possibility to differentiate between natural and anthropogenic NPs based on multi-metal fingerprints has received considerably interest.^25–28,47^ However, as an inventory of natural and anthropogenic particle types is currently lacking, NP identification based on database searching or spectral matching approaches is not possible. To identify different clusters of mmNPs, which may be assigned to specific sources, we performed clustering analyses as described in the methods section. Our sp-ICP-TOFMS analyses revealed substantial differences in the numbers of mmNPs detected in the influent and effluent samples from the different WWTPs. For some WWTP samples, such as W1, more than 25% of the NPs are multi-metal. With these high mmNP fractions, significant information content on the description of NPs populations entering and exiting WWTPs is associated with the composition of mmNPs. In Fig. 4a, we present results from hierarchical clustering analysis of the mmNP data from the influent of WWTPs sampled in Nov. 2019. Results from clustering analysis of sp-ICP-TOFMS data from the May 2019 samples are presented in Fig. S8.†

In Fig. 4a, the major clusters from each WWTP are “leaves” in the cluster tree and are represented with a name that corresponds to the wastewater sample and the most frequent elements in the mmNPs of that cluster. The branches of the cluster tree show the relation of mmNP clusters across the different WWTPs. Inter-sample clusters that have a distance of 0.5 or greater are considered distinct; overall, we identify 23 mmNP clusters across the five WWTP samples. The dendrogram also reveals that some mmNP clusters are conserved across all WWTPs (*i.e.* have branches from all WWTPs [I1–I5]) and other clusters are only present in one or a few WWTPs. Our hierarchical clustering scheme reduces mmNP data into just 23 clusters and 77 leaves; however, the original data from each of these mmNPs is still accessible. Cluster tree representation offers the possibility to select branches or leaves of interest, and then extract and examine mmNP data from that subset. For example, one can evaluate the PNCs or element masses per particle for any given branch or leaf. For the remainder of this manuscript, we explore selected aspects of the mmNP dataset that become apparent through clustering analysis.

The dendrogram in Fig. 4a shows that some clusters of mmNPs are more conserved than others: mmNP types that exist in all samples include those rich in Ti–Zr, Zn–Cu, Au–Ag, Ce–La, and Zr–Y. These mmNPs are found in both the May and Nov. measurements. The ubiquity of these mmNP types suggests a natural origin, or at least common anthropogenic sources: if mmNPs were from specific point sources of anthropogenic NP pollution, they would likely be found only in selected WWTPs. In addition, we know from previous research that our mmNP types match, at least in part, results from studies focused on Ce, Ti, Zr, and Zn natural NPs.^14,15,22,24–29^ In Fig. 4b–g, we extract data from selected mmNP clusters and plot the normalized mass of elements that are measured in at least 1% of the particles that make up each clusters. Normalization was done at the particle level: the quantified masses of each element were divided by the mass of the most frequently occurring element from the cluster. For example, in Fig. 4b–g, the normalizing elements were Ti, Zn, Ag, V, Ce, and Zr, respectively, because these elements were detected most often in particles that make up the clusters. The rather well conserved element ratios in these mmNP clusters further emphasizes that these mmNP clusters likely originate from comparable sources. In Table 2, we report the number of NPs measured, the number fractions, and the PNCs of each of the mmNP types reported in Fig. 4b–g. Of all the major clusters of mmNPs, we find most variance of the element ratios for the Ti–Zr mmNP type. This finding again supports previous research results in which the element fingerprints of Ti-rich naturally occurring NPs most often contain Al, Zr, and Nb, with rare instances of Ta and Zn association. Our data also match what might be expected from geochemistry, as it is known that titanium oxide (*e.g.* rutile, TiO_2_) has common associations with Nb, Ta, and W, among others.^33,34^ The Zr–Y rich cluster (see Fig. 4g) also has a well-defined element distribution that matches the expected fingerprint of naturally occurring zircon (ZrSiO_4_), which has associations with Hf, Th, U, Y and rare earth elements.^48,49^ In Fig. S9,† we present expanded inter-sample HCs with the median masses of elements in each of the representative mmNP proxies, as well as number concentrations.

**Table 2:** Number of NPs measured, number fractions, and the PNCs of the mmNP types reported in Fig. 4b–g. Note that samples I3–I5 were analyzed at a 10-times dilution and I1–I2 at 100-times dilution. Each of the samples were analyzed for ∼450 s by sp-ICP-TOFMS. PNCs refer to concentration in original sample. Number fraction is the number of NPs of a particular mmNP type divided by the total number of NPs measured in a sample

	Ti–Zr	Zn–Cu	Au–Ag	Bi–V	Ce–La	Zr–Y
	Number of detected particles					
I5	2320	2637	14		970	220
I4	3946	378	6		134	56
I3	3786	916	38	137	2442	179
I2	211	113	2		56	29
I1	307	323	2	4	4642	34

	Number fraction (%)					
I5	2.72	3.09	0.02		1.14	0.26
I4	19.09	1.83	0.03		0.65	0.27
I3	6.51	1.58	0.07	0.24	4.2	0.31
I2	3.24	1.74	0.03		0.86	0.45
I1	1.51	1.59	0.01	0.02	22.81	0.17

	PNC (particles per mL)					
I5	2.1 × 10^5^	2.4 × 10^5^	1.3 × 10^3^		8.7 × 10^4^	2.0 × 10^4^
I4	3.7 × 10^5^	3.6 × 10^4^	5.7 × 10^2^		1.3 × 10^4^	5.3 × 10^3^
I3	4.8 × 10^5^	1.2 × 10^5^	4.8 × 10^3^	1.7 × 10^4^	3.1 × 10^5^	2.3 × 10^4^
I2	2.2 × 10^5^	1.2 × 10^5^	2.1 × 10^3^		5.9 × 10^4^	3.0 × 10^4^
I1	3.1 × 10^5^	3.3 × 10^5^	2.0 × 10^3^	4.1 × 10^3^	4.8 × 10^6^	3.5 × 10^4^

In addition to ubiquitous NNPs, hierarchical clustering also identifies rather exotic particle types including NPs rich in Au–Ag, Rh–Pd, Bi–V–(Mo), Sb–W, Cr–Ni, Zn–Cu, Mn–Cu, and Ba–La. We find a unique Bi–V-rich mmNP cluster in two of the WWTP influent samples. As shown in Fig. 4e, the mean mass ratios of Bi : V in this cluster are 5 in I3 and 6.8 in I1. Because ^209^Bi is about four-times heavier than ^51^V, the measured element ratios are reasonably consistent with a one-to-one atomic ratio (4.1 : 1, Bi : V mass ratio) of bismuth vanadate (BiVO_4_), especially considering the low numbers of particles recorded. Bismuth vanadate—which is sometimes doped with molybdenum (as also found in the Bi–V–Mo cluster from I3)—is used as yellow pigment in industry^50^ and as a catalyst,^51^ and therefore is very likely anthropogenic in origin. BiVO_4_ synthetic NPs have been previously measured by sp-ICP-TOFMS;^52^ however, to the best of our knowledge, have never been observed as a contaminant in a wastewater or environmental sample. Another unexpected mmNP class found by hierarchical clustering is the fairly well conserved Au–Ag cluster (Fig. 4d) that contains 5–10 times more Au than Ag on a mass basis. We find the Au–Ag cluster in all WWTP samples from both of our collection days. These NPs may originate from gold alloys used in the jewelry manufacturing industry, and thus are likely anthropogenic in origin.

### Analysis of sub-cluster of Ce–La particles

**Fig. 5:**
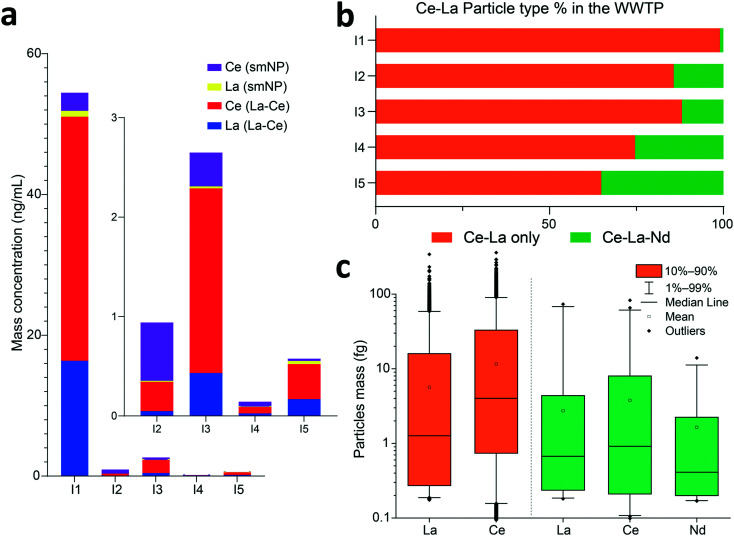
Further insight into the Ce–La mmNP cluster. a) Comparison of Ce and La as smNPs and mmNPs in terms of mass concentrations in I1–I5. Insert graph shows the I2–I5, which have substantially lower mass concentrations of Ce and La compared to I1. b) Comparison of the PNCs of Ce–La and Ce–La–Nd mmNP types. c) Comparison of Ce and La mass distributions in the two sub-types of mmNPs found in sample I1. The Ce–La mmNP type has a higher Ce : La mass ratio of ∼3.5, whereas the Ce : La mass ratio in Ce–La–Nd mmNPs tends to center around 2.

Hierarchical clustering analysis provides a useful means to reduce mmNP data into major clusters; however, as the complexity of a system increases, it becomes difficult to decide at which distances clusters should be defined. This limitation might be overcome by using machine learning approaches.^25,53^ An alternative approach is to evaluate sub-clusters within our analysis. For example, in Fig. 4a, the Ce–La cluster appears to be bifurcated into two distinct sub-clusters: those with Ce–La–Nd (from I2–I5) and just Ce–La (from I1) major-element signatures. Upon closer examination of Ce and La NP signals, we find that, in terms of mass concentration, Ce and La mostly exist together rather than as smNPs in all the WWTP samples (see Fig. 5a and S10a†). However, the Ce–La containing mmNPs found in the WWTPs have different associated elements. For example, in Fig. 5b, we plot the percentages (in terms of particle number) of mmNPs that contain Ce–La only and those that have detectable quantities of Ce–La and Nd. We find that WWTP 1 has a significantly lower fraction of Ce–La–Nd particles compared to Ce–La particles than the other WWTPs. In Fig. 5c, we plot the mass distributions of Ce, La, and Nd in both the Ce–La and Ce–La–Nd mmNPs found in the I1 sample. The Ce–La only mmNPs found in I1 actually have higher median masses of Ce and La compared to the Ce–La–Nd mmNP type, which indicates that the identification of Ce–La mmNPs is not due to detection of a small-particle subset of a broader Ce–La–Nd particle type. If Nd were present at similar mass fractions in the Ce–La mmNPs, then this Nd would have been detected. This is further emphasized by considering the Nd detection limit (see ESI† Excel sheet) for Nd in sample I1, which is 0.15 fg—well below the mass of Nd that we would expect from Ce–La–Nd mmNPs with median Ce mass of ∼3 fg.

The Ce–La mmNPs found in sample I1 represent a distinct class of mmNP that is demonstrably different that the Ce–La-containing mmNPs in samples I2–I5. Combined with the fact that these Ce–La mmNPs were measured at about ten-times higher concentration in sample I1 (both in terms of mass concentration and PNC), this suggests that these Ce–La only mmNPs come from a unique source that could be anthropogenic. Our finding indicates that measurement of La and Ce alone could be insufficient to classify Ce-containing NPs as natural or anthropogenic in origin. The sub-class of Ce–La-only NPs did not separate by our initial clustering analysis because the La-to-Ce ratios in these NPs are not significantly different, as shown in Fig. S11.† Our results are consistent with those of a recent study,^54^ which reports the measurement of high industrial contribution of Ce to the sludge of W1 based on REE ratios of the sludge compared to that of local soils. Additionally, recent X-ray absorption spectroscopy analysis of Ce–La NPs from W1,^23^ also suggest that Ce–La association is not a definitive marker for naturally sourced Ce-containing NPs. The source of the Ce–La-only NPs in I1 is currently unknown and is under investigation, but it could be related to glass industry^55^ or other technical industries in the area of W1. In the extraction of Nd from REE ores, it is common practice separate Ce + La as a mixture from other REEs,^44^ so anthropogenic Ce–La only NPs are reasonable. Moreover, the Nd-depleted characteristics of the Ce–La mmNPs do not match expectations for common Ce-bearing minerals such as allanite, monazite, or bastnäsite.

A more definitive assignment of the anthropogenic or natural origin of the Ce–La mmNPs might, in principle, be possible with transmission electron microscopy (TEM) combined with energy dispersive X-ray spectroscopy (EDX) analysis based on morphological features combined with elemental composition. However, TEM-EDX measurements have to be conducted manually by operators, as automated TEM-EDX solutions are still in their early stage of development.^19^ Without automated analysis (and excluding sample preparation and instrument tuning), a good rule of thumb would be that, for TEM-EDX, a best-case particle analysis rate would be ∼1 minute per particle. In sample I1—which has the highest concentration of Ce–La NPs by far—22.8% of the measurable particles we record are Ce–La containing NPs. Thus, if TEM-EDX analysis were performed for 1 hour, we would expect to measure *at most* 14 Ce–La containing NPs. In contrast, for this sample, 4642 Ce–La particles were measured in just 7.5 minutes by sp-ICP-TOFMS. Moreover, there are likely many particles (*e.g.* particles below size detection limits and silicate, clay, or organic NPs) that are undetectable by sp-ICP-TOFMS, but would interfere with TEM analysis and further reduce the probability of measuring representative samples of specific mmNPs. The chance of finding mmNP types that exist at low number concentrations and at number fractions <1% are very low for TEM. TEM-EDX is currently not a viable approach for element composition determination of particles in complex particle-rich environmental samples. Single particle ICP-TOFMS provides the unique capability to simultaneously evaluate widely variable number concentrations of many populations of smNPs and mmNPs and to identify particle clusters (*i.e.* types) based on correlation within elemental compositions of the measured NPs.

## Conclusions

We presented a new strategy for the analysis of NPs in environmental samples. From wastewater samples that likely have a mix of different types of natural and anthropogenic NPs, sp-ICP-TOFMS produces a rich data set composed of a broad range of smNPs and mmNP types. To break down the inherent complexity of our sp-ICP-TOFMS datasets and reduce the dataset to an interpretable form, we combined online microdroplet calibration with an automated data processing system. With this approach, we are able to extract NP data from raw ICP-TOFMS time traces, determine the element masses in single NPs, record PNCs, correct the data sets for the presence of particle-coincidence events, and cluster mmNPs in an unsupervised fashion. Until now, a major bottleneck in sp-ICP-TOFMS analysis was data processing. However, we are now able to rapidly deliver multi-element quantitative analysis of NP populations in real samples, and to provide initial clustering analysis to aid the interpretation of the data—for example, through in the identification of unique mmNP types.

As case study, we presented a multiplexed analysis of metal and metalloid-containing NPs in wastewater samples from five WWTPs across Switzerland. Wastewater analysis is a complex case because it has inputs of municipal, industrial, and environmental origins. In our study, we found more than 30 different types of smNPs at number concentrations ranging from 3 × 10^2^ to 4 × 10^6^ particles mL^−1^ in the influent and effluent of the WWTPs. We also found that mmNPs are prominent in many of the WWTPs, making up as much as 27% of the total particle number. Our study indicates high removal efficiencies of the WWTPs for both smNPs and mmNPs based on total mass and PNC for different elements and mmNP types. In general, NP removal efficiencies based on particle mass concentrations are higher than those based on number concentration, which suggests that larger NPs are, generally, retained to a greater extent in the WWTPs than smaller NPs. Through an unsupervised hierarchical cluster analysis, we identified 23 major unique mmNP types in the influent of the WWTPs. At least five of these mmNP types are present in all WWTP samples, including mmNPs rich in Ti–Zr, Zn–Cu, Ag–Au, Ce–La, and Zr–Y. In our analysis, we looked in more detail at the Ti–Zr, Zn–Cu, Ce–La, and Zr–Y mmNP types and found comparable element mass ratios within individual NPs in all wastewater samples, which points toward a natural origin of these mmNP types. On the other hand, hierarchical clustering also uncovered several particle types present in just a few wastewater samples. These uniquely occurring NP types may originate from specific point sources and likely represent anthropogenic NP types. Examples, of such NP types include Bi–V and Rh–Pd NPs, which have not been reported from environmental / wastewater samples to date. Further, our data suggest that, in at least one influent sample, there is a sub-class of Ce–La-only mmNPs; this particle class is distinct from Ce–La–Nd mmNPs and may be of anthropogenic origin. A key feature of our measurement approach is that it enables the detection and preliminary classification of both expected and unexpected or unforeseen NP types. Here, we presented our results as an exemplar of particle-screening analysis by high-throughput sp-ICP-TOFMS. Such screening analysis is essential to expand our understanding of particle types—both anthropogenic and natural—and abundances in environmental compartments. As such, sp-ICP-TOFMS is attractive tool to refine mass-transport models, to monitor nano-pollution, and also to discover novel particle targets.

Interpretation of our sp-ICP-TOFMS data set in terms of NP origin is an ongoing process and we expect continued refinement of our understandings. In fact, the continued refinement of our knowledge about mmNP fingerprints (both of natural and anthropogenic NP types) and the expected types of mmNPs in various environmental compartments is a substantial benefit of our current measurement approach. Because our approach allows for both high throughput data collection and data analysis, we are now able to expand our inventory of NPs—which will, in turn, improve our classification models. An eventual aim of such studies is to establish a database approach for the identification of different NP types. In this vein, we anticipate the expanded use of high-throughput sp-ICP-TOFMS analysis for the continued exploration of the presence both of natural and anthropogenic NPs in environmental systems.

## Conflicts of interest

There are no conflicts to declare.

## Supplementary Material

EN-008-D0EN01066A-s001

EN-008-D0EN01066A-s002
