# Supplementary material for: Emerging investigator series: automated single-nanoparticle quantification and classification: a holistic study of particles into and out of wastewater treatment plants in Switzerland
Source: Environ Sci Nano. 2021 Mar 23;8(5):1211–25. doi: 10.1039/d0en01066a (PMC8136323; doi:10.1039/d0en01066a)
Supplement: EN-008-D0EN01066A-s001 [file EN-008-D0EN01066A-s001.pdf]

Electronic Supplementary Material (ESI) for Environmental Science: Nano.
This journal is © The Royal Society of Chemistry 2021

Supporting Information for *Automated Single-Nanoparticle Quantification and Classification: A Holistic Study of Particles into and out of Wastewater Treatment Plants in Switzerland*

Kamyar Mehrabi[1], Ralf Kägi[2], Detlef Günther[1], Alexander Gundlach-Graham[3*]

1. Department of Chemistry and Applied Biosciences, ETH, Zurich, Switzerland,

2. Department of Process Engineering, Eawag, Dübendorf, Switzerland

3. Department of Chemistry, Iowa State University, Ames, USA

*correspondence: alexgg@iastate.edu

Table of Contents

Table S1	Operating parameters
Table S2	List of selected elements and the isotopes used for sp-ICP-TOFMS analysis
Table S3	Single-particle critical value expression parameters
Figure S1	WWTP Sampling sites
Figure S2	Response curve of the instrument
Figure S3	Example of hpCC for Ag-Sn mmNPs
Figure S4	Graphical illustration of the two stage hierarchical clustering
Figure S5	Mass distributions of all elements
Figure S6	Average equivalent spherical diameter detection limits of oxide form of elements
Figure S7	smNPs and mmNPs detection across different WWTPs (May 2019)
Figure S8	Dendrogram of clustered multi elemental particles (May 2019)
Figure S9	Representation of all mmNP clusters in influent samples I1 to I5
Figure S10	Insight into the Ce-La cluster (May 2019)
Figure S11	Comparing the La to Ce mass ratio of the particle events

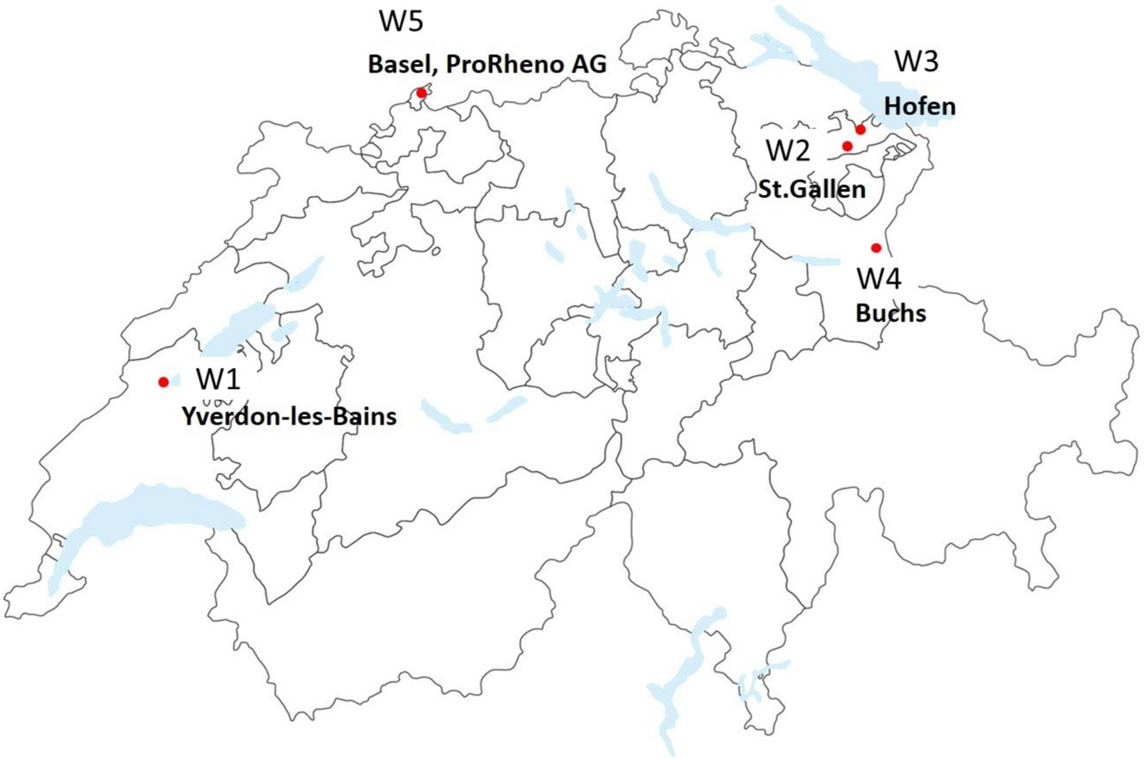

Fig. S1. WWTP Sampling sites. For each site, the respective locality as well as the abbreviation for it in the main text are highlighted. The figure was reproduced from Gogos et al.[1]

Table S1. Operating parameters.

**Microdroplet Introduction**	
Droplet Diameter	65 µm
Droplet Frequency	50 Hz
Used Droplet Burst Length	750 droplets, 15 s
He Gas Flow in Falling Tube	0.56 L min^{-1}
Ar Gas Flow in Falling Tube	0.15 L min^{-1}
**Pneumatic Nebulizer**	
Nebulizer Gas (Ar)	0.9 L min^{-1}
Solution Uptake Rate	~100 µL min^{-1}
**ICP Conditions**	
Intermediate Gas Flow (Ar)	0.8 L min^{-1}
Outer Gas Flow (Ar)	15 L min^{-1}
Power	1550 W
Sampling Position	4.5 mm above load coil

Collision/Reaction Cells		
He Gas Flow	2.0 mL/min	
H_2 Gas Flow	0.5 mL/min	

Notch filter Conditions	m/q	V
Notch 1	18.0	1.450
Notch 2	22.0	0.920
Notch 3	30.0	0.870
Notch 4	38.0	0.970

TOFMS Conditions	
TOF Spectral Acquisition Rate	21.739 kHz
Averaged Spectrum Acquisition Rate*	494.07 Hz (2.02 ms)

*Averaged mass spectra are composed of data summed from 44 full mass spectra.

Table S2. List of selected elements and the isotopes used for sp-ICP-TOFMS analysis. When multiple isotopes were used for measurement of an element, the MS-intensity time traces of those isotopes were summed prior to finding of NP signals. Elements in bold red are those present in the microdroplet standard solution used to establish element sensitivity factors.

Element	Concentration in droplet (ng/mL)	Measured Isotope(s)			Element	Concentration in droplet (ng/mL)	Measured Isotope(s)		
**Al**	203	[27Al]			Sb	--	[121Sb]	[123Sb]	
**Ti**	202	[47Ti]			**Cs**	19.9	[133Cs]		
V	--	[51V]			Ba	--	[137Ba]		
Cr	--	[52Cr]			La	--	[139La]		
**Fe**	101	[57Fe]			**Ce**	20.7	[140Ce]		
Mn	--	[55Mn]			Nd	---	[144Nd]	[146Nd]	
Ni	--	[60Ni]			Er	--	[166Er]		
**Cu**	101	[63Cu]	[65Cu]		Hf	--	[178Hf]		
**Zn**	101	[64Zn]	[66Zn]		Ta	--	[181Ta]		
Y	--	[89Y]			W	--	[182W]	[184W]	[186W]
Zr	--	[90Zr]			**Pt**	102	[194Pt]	[195Pt]	[196Pt]
Nb	--	[93Nb]			**Au**	103	[197Au]		
Mo	--	[95Mo]	[97Mo]		Tl	--	[205Tl]		
Rh	--	[103Rh]			**Pb**	20.2	[206Pb]	[208Pb]	
Pd	--	[105Pd]			Bi	--	[209Bi]		
**Ag**	20.2	[107Ag]	[109Ag]		Th	--	[232Th]		
**Sn**	103	[118Sn]	[120Sn]		**U**	2.01	[238U]		

Table S3. Single-particle critical value ($L_{c,sp}$) line formulae for different false positive rates (alpha values) based on compound-Poisson modelling of the measure single-ion-signal histogram from the ICP-TOFMS instrument.[2]

$L_{c,sp}$		
alpha	Slope	Intercept
0.005	2.7	1.5
0.002	3.2	1.5
0.001	3.4	1.9
0.0005	3.8	2.0
0.0002	4.0	2.4
0.0001	4.2	2.6
0.00005	4.5	2.8
0.00002	4.7	3.2
0.00001	4.9	3.3

Semi-quantitative mass analysis

As shown in equation S1, the absolute sensitivity of each element (S) is calculated, where I is the droplet signal, A is the abundance of used isotope(s), m is the mass of element of interest in the droplet and MW is the molecular weight of the element in the droplet. This formula used to calculate for the absolute sensitivity of the element in the droplet and further predict the ones missing as shown in Fig S1.

$$S = \frac{I/A}{m/MW} \quad \textbf{(S1)}$$

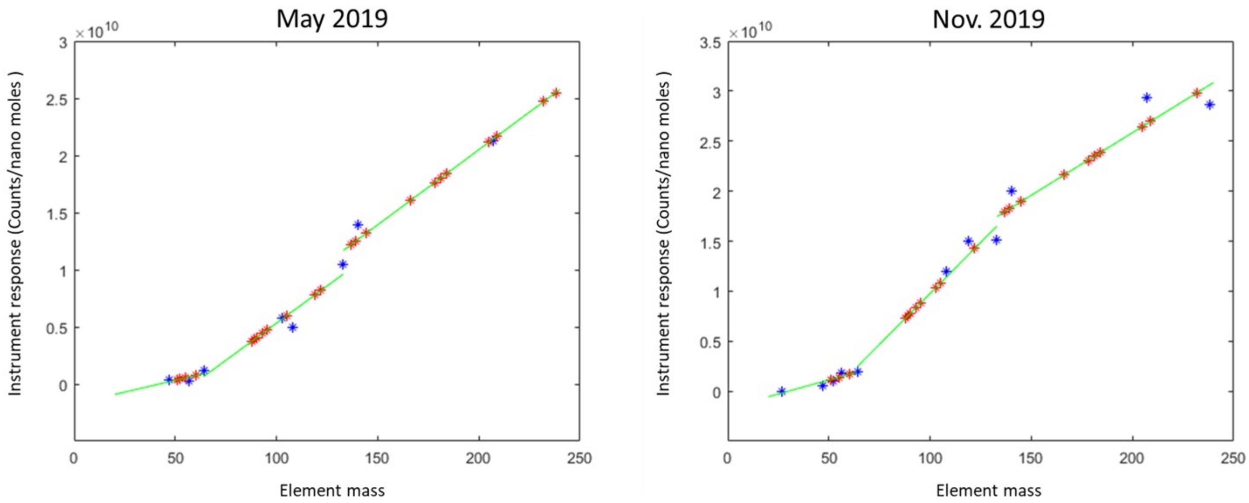

Fig. S2. Response curves of the ICP-TOFMS instrument for the May and November 2019 samplings. The elements present in the microdroplet standard are in blue and predicted sensitivities of elements of not

in the droplet solutions are in red. The green line shows the linear regression of the known response in each low, median, and high mass region.

Hetero-particle coincidence correction (hpCC)

The first step in our hpCC algorithm is to group every mmNP that have identical element fingerprints and sort the fingerprints from those with the most to those with the fewest number of elements. Starting with the fingerprint with the most elements, we define this particle fingerprint (f_t) as its representative set of elements, as shown in equation S2, in which $E_1, E_2, \ldots E_m$ are the fingerprint elements. Next, from our pool of found smNP and mmNP types we find possible combinations of two precursor particles (f_1, f_2) that together would produce a coincident particle with all fingerprint elements in f_t (see equation S3), and in which no fingerprint elements are conserved between f_1 and f_2 (see equation S4). Once these f_1 and f_2 fingerprints are identified, we predict number of coincident particles based on equation S5, in which $N_{f_1}$and N_{f_2} are the number of detected events with f_1 and f_2 fingerprints, N_{total} is the total measurement data points, and $C_{(f_1, f_2)}$ is predicted number of coincident $f_1$and f_2 events.

$$f_t = \{E_1, E_2, \ldots E_m\} \quad \textbf{(S2)}$$

$$(f_1, f_2) \subseteq f_t \quad \textbf{(S3)}$$

$$f_1 \cup f_2 = f_t \,, f_1 \cap f_2 = \emptyset \quad \textbf{(S4)}$$

$$\frac{N_{f_1} * N_{f_2}}{N_{total}} = C_{(f_1, f_2)} \quad \textbf{(S5)}$$

If the $C_{(f_1, f_2)}$ is greater than 1, then we attempt to identify which of the measured particles with element signature f_t are likely due to particle coincidence (i.e. f_1 and f_2 occurring together) rather than due to true multi-metal composition. To accomplish this, we define a hypothetical selection criterion and a scoring method based on the ratios of element signals in the measured mmNPs with composition f_t. Let us consider the set of individual particle signals that all have the identical fingerprint, f_t, to be $X_{f_t} = \left\{x_1, x_2, \ldots x_{N_{f_t}}\right\}$. For scoring individual particle signals, we first find the element with the largest median signal (*E*$_{max}$) in the set of particle signals, X_{f_t}, as shown in equation S6. To normalize the element signals within each particle, we then divide the element signals of all elements in each particle by the signal of the *E*$_{max}$ in the particle, i.e. $E_{k_{c_j}} / E_{Max_{c_j}}$. A sole normalized value for each particle in the set C_{f_t} is defined as $Norm_{x_j}$ and is the sum of the normalized element signals in each particle, as shown in equation S7. A score of how similar each particle is to the overall group (S_{x_j}) is defined as the distance of the particle's $Norm_{x_j}$ from the median $Norm_{X_{f_t}}$ values of the set, X_{f_t}, as shown in equation S8. Particles with the largest S_x values are least likely to be part of the true mmNP set and are therefore identified as particle coincident events. A total of $C_{(f_1, f_2)}$ particles with the highest S_x scores are then removed from the X_{f_t}

set and broken down into their composite f_1 and f_2 particles. In Fig S3, we provide an example of mmNP similarity scoring and the subsequent breakdown of Ag/Sn mmNP signals into coincident and non-coincident events. Once all possible coincident particle combinations and their coincidence probabilities are calculated for particles with a given set of fingerprint elements, i.e. f_t, the hpCC process is repeated for mmNP signals with successively fewer numbers of fingerprint elements. hpCC is complete when all unique mmNP signals are divided into coincident particle and non-coincident particle fractions.

$$E_{Max} = Max(Median(E_i), i \in f_t)) \quad \textbf{(S6)}$$

$$Norm_{x_j} = \sum_{k=1}^{m} \left(E_{k_{x_j}} \middle/ E_{Max_{x_j}} \right) \quad \textbf{(S7)}$$

$$S_{x_j} = |Norm_{x_j} - Median(Norm_{X_{f_t}})| \quad \textbf{(S8)}$$

Example results from hpCC are shown in Fig S3. Hetero-particle coincident events occur when two or more non-identical discrete particles are present in the plasma simultaneously; the likelihood of these events occurs follows Poisson statistics. Through sample dilution one can significantly reduce the chance of coincidence: If a sample is diluted "d" times then all nanoparticles of interest are also diluted "d" times; however, the probability of a coincident event reduces as the square of the dilution factor, i.e. as "d^2", see equation S11. Because coincident NP events are reduced more rapidly than individual NP events, we can use dilution experiments to check the efficacy of our hpCC algorithm to assess whether the predicted true mmNP are also present in the diluted sample. Likewise, we can assess whether the predicted coincident events are absent in the diluted sample.

$$P_{A \cap B} = P_A \times P_B \quad \textbf{(S9)}$$

$$P_{A'} = P_A.d \ , \ P_{B'} = P_B.d \quad \textbf{(S10)}$$

$$P_{A' \cap B'} = P_A.d \, .P_B.d = P_{A \cap B}.d^2 \quad \textbf{(S11)}$$

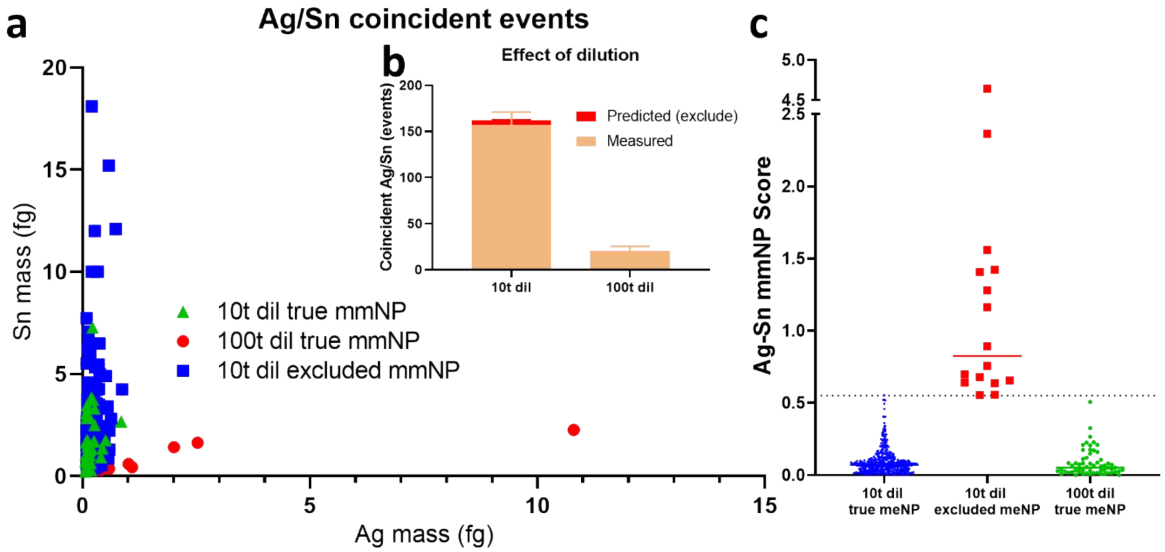

Fig. S3. Example of hpCC for Ag-Sn mmNPs **. a**)The scatter plot shows the Ag-Sn coincident events at two dilutions, where green triangles are true mmNPs found in the higher dilution (100-times dilution) sample, blue rectangles are selected as true mmNPs in the 10-times dilution and red circles are selected as coincident events of Ag and Sn single-metal particles. **b**) the measured and predicted number of Ag-Sn coincident events (at 100x dilution, no coincident events were predicted). **C)** Scores of each individual Ag-Sn mmNP; particles that had scores more than 0.54 are excluded as coincident particle events.

In Fig. S3, we plot the masses of Ag and Sn in multi-metal signals at two different dilutions. At a dilution factor of just 10-times, we predict ~5 % of the multi-metal signals are the result of coincident particle events. Based on equations 6-8 for selection criteria, we identify a subset of the measured multi-metal NPs that are likely to be coincident particle events and then exclude these signals from the pool of true multi-metal Ag-Sn NPs. With 100-times dilution, we predict no coincident particle events between Ag and Sn: all Ag-Sn events present in the 100-times dilution sample are likely true mmNPs. Through comparison of scatter plot of the masses of Ag vs Sn in individual measured particles, we demonstrate that our selection criteria used to decide which multi-metal signals are "true" and which are "false" is performing as expected. Our analysis of measured Ag-Sn indicates that high Ag:Sn ratio signals are likely false and this is also found in the 100-time dilution samples. No true mmNPs with Ag mass above 1 fg are found, just as predicted. Importantly, hpCC enables improved measurement statistics for mmNPs because it allows for higher number of events to be counted per unit time.

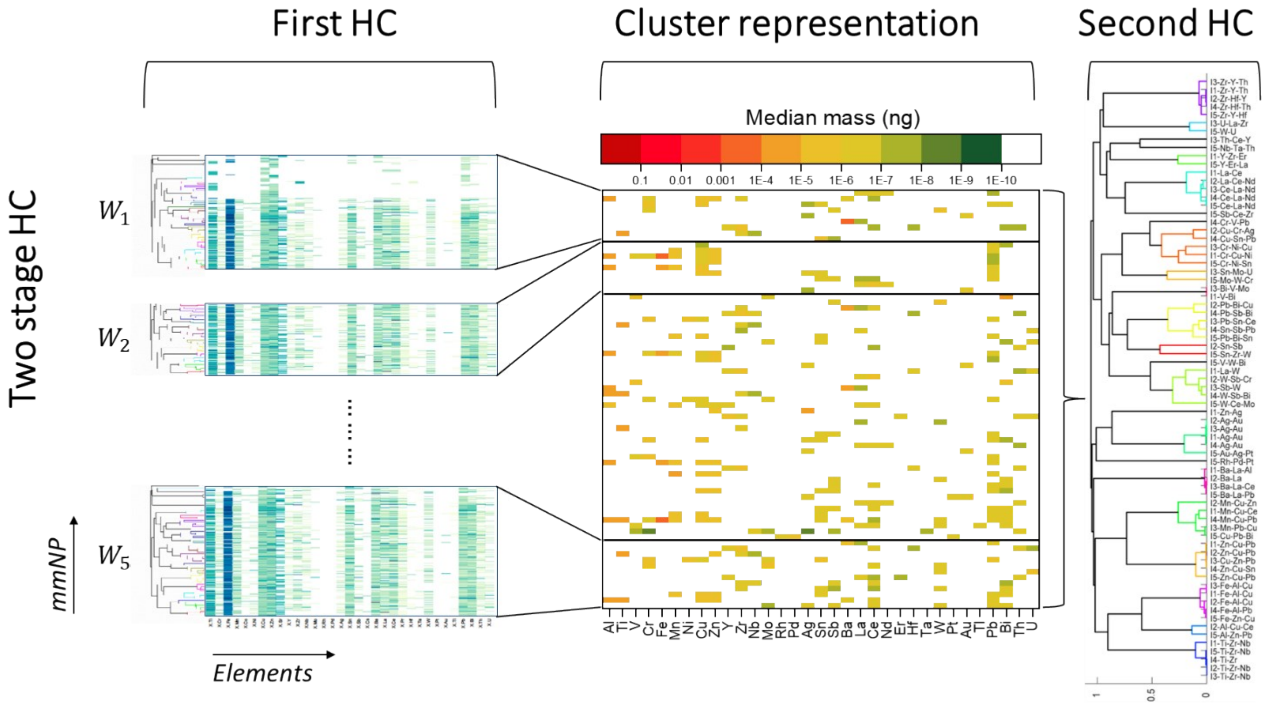

Fig. S4. Graphical illustration of the two-stage hierarchical clustering (HC) of mmNPs. In the first step, HC is performed on individual samples. Then, a representative for each cluster from each sample was taken as described in the manuscript. These representative mmNP types are clustered again to find inter-sample clusters and unique mmNP types.

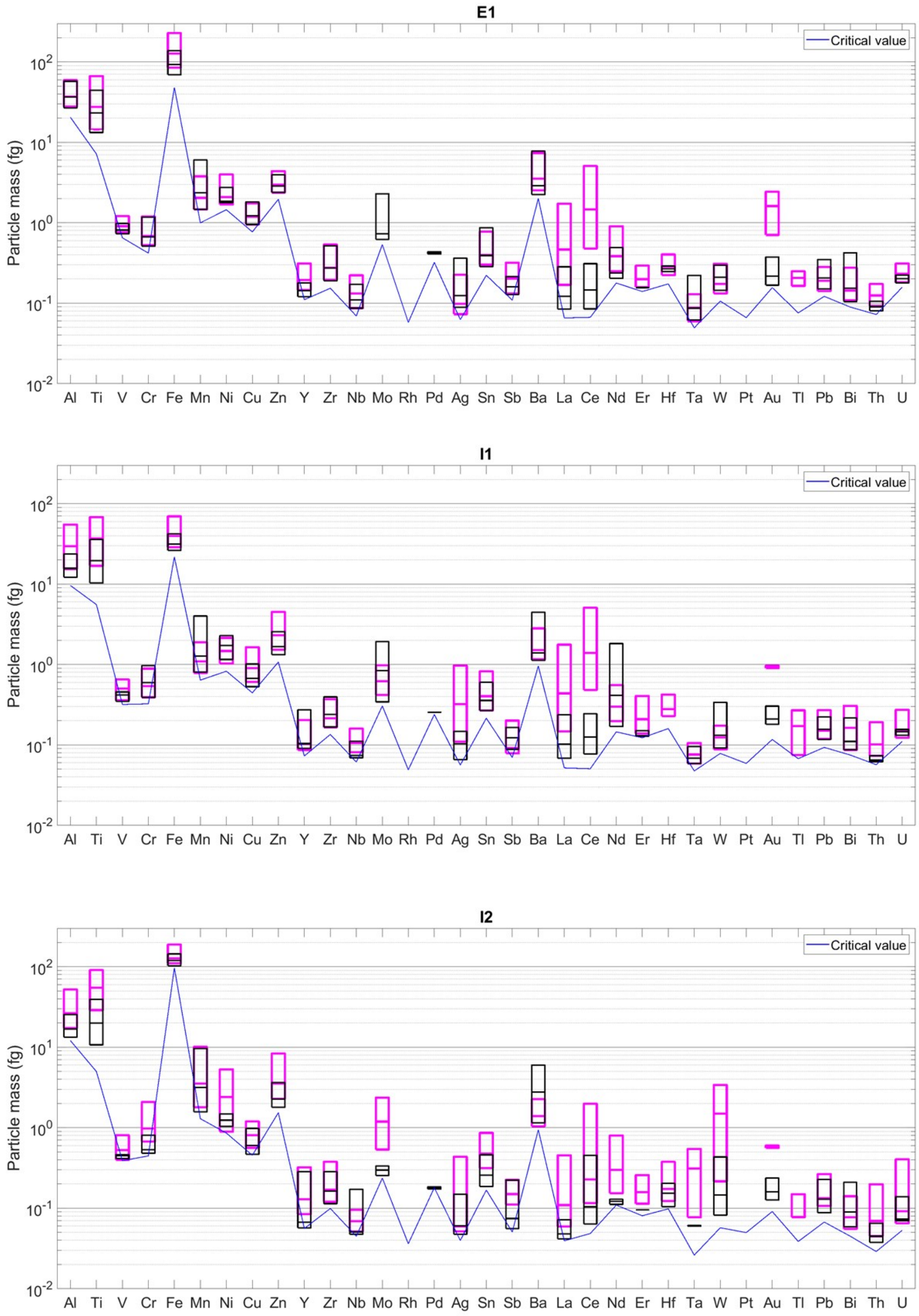
E1
I1
I2
Critical value
Particle mass (fg)
10^{-2}
10^{-1}
10^{0}
10^{1}
10^{2}
Al Ti V Cr Fe Mn Ni Cu Zn Y Zr Nb Mo Rh Pd Ag Sn Sb Ba La Ce Nd Er Hf Ta W Pt Au Tl Pb Bi Th U

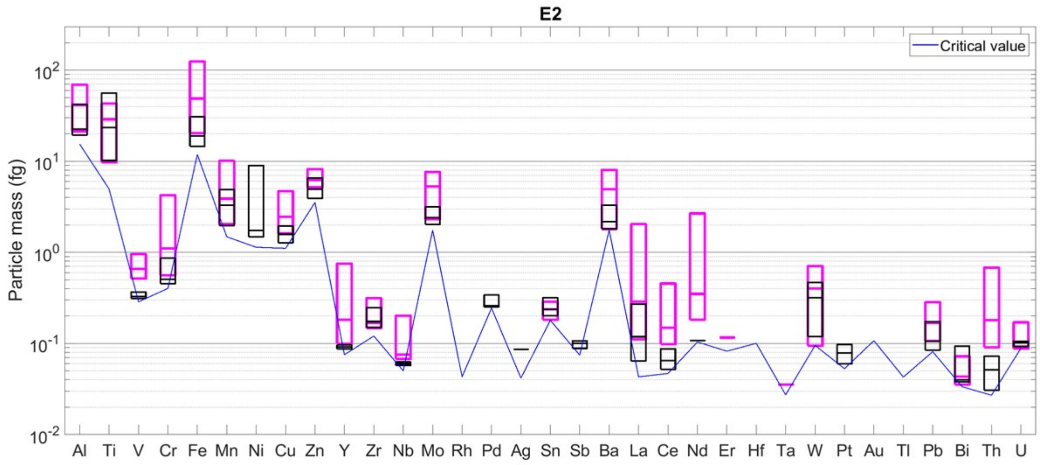
E2
Critical value
Particle mass (fg)
10^2
10^1
10^0
10^{-1}
10^{-2}
Al Ti V Cr Fe Mn Ni Cu Zn Y Zr Nb Mo Rh Pd Ag Sn Sb Ba La Ce Nd Er Hf Ta W Pt Au Tl Pb Bi Th U

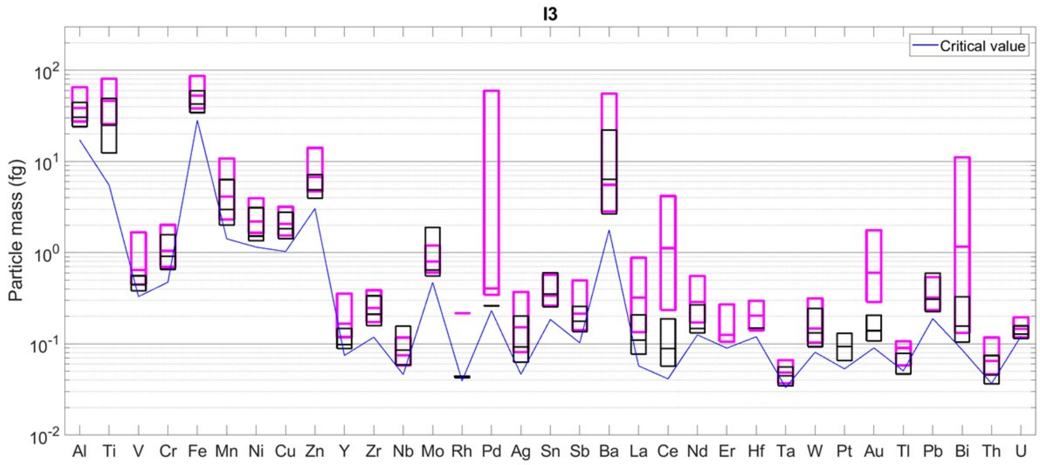
I3
Critical value
Particle mass (fg)
10^2
10^1
10^0
10^{-1}
10^{-2}
Al Ti V Cr Fe Mn Ni Cu Zn Y Zr Nb Mo Rh Pd Ag Sn Sb Ba La Ce Nd Er Hf Ta W Pt Au Tl Pb Bi Th U

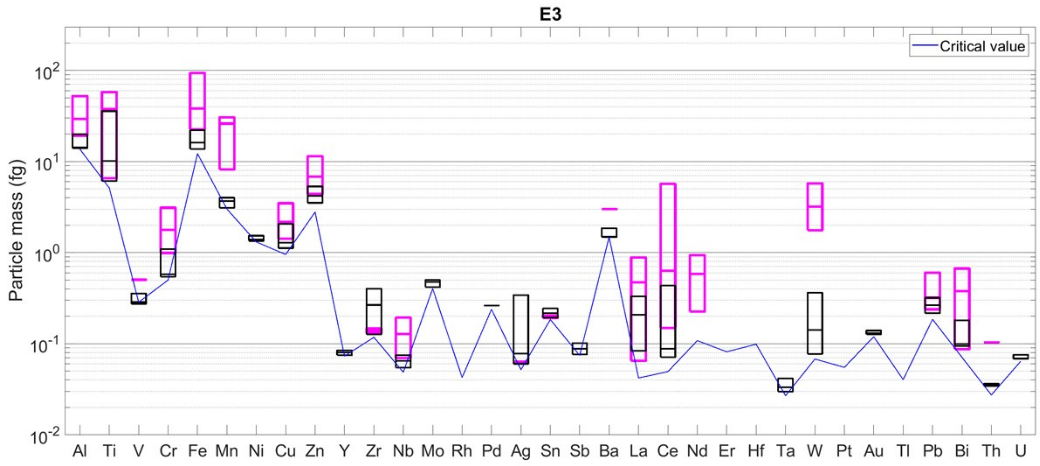
E3
Critical value
Particle mass (fg)
10^2
10^1
10^0
10^{-1}
10^{-2}
Al Ti V Cr Fe Mn Ni Cu Zn Y Zr Nb Mo Rh Pd Ag Sn Sb Ba La Ce Nd Er Hf Ta W Pt Au Tl Pb Bi Th U

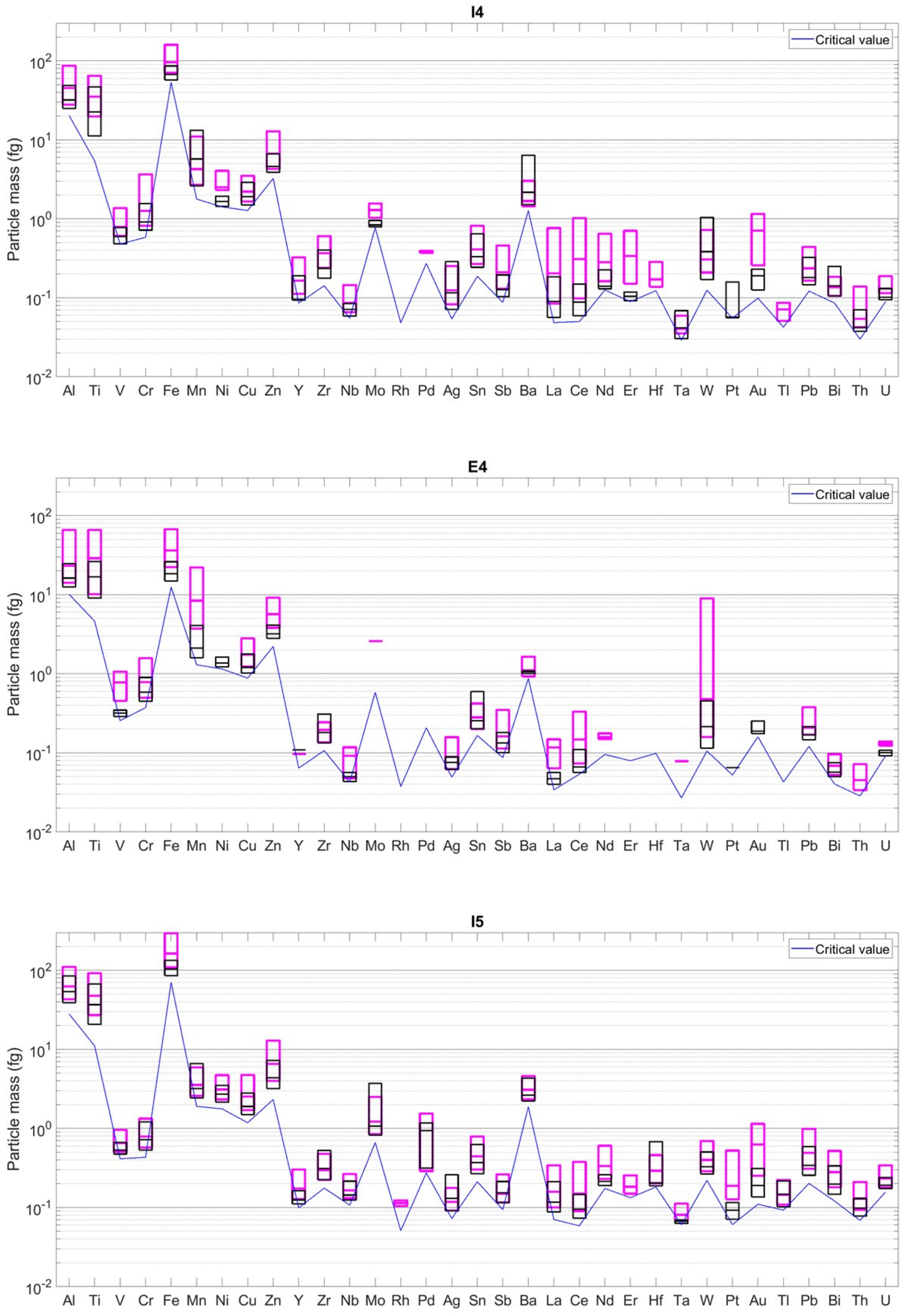
I4
E4
I5
Critical value
Particle mass (fg)
10^2
10^1
10^0
10^-1
10^-2
Al Ti V Cr Fe Mn Ni Cu Zn Y Zr Nb Mo Rh Pd Ag Sn Sb Ba La Ce Nd Er Hf Ta W Pt Au Tl Pb Bi Th U

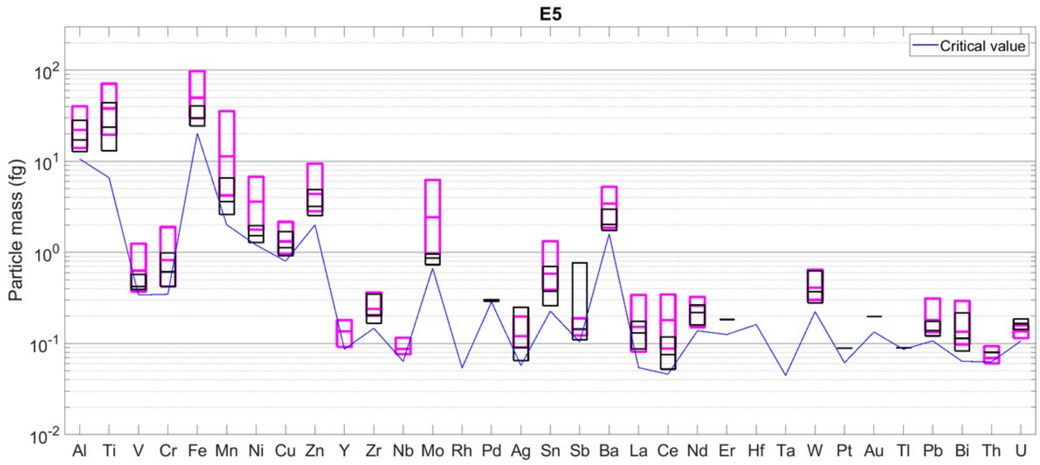

Fig. S5. Mass distributions of all elements in influent (I) and effluent (E) samples, plotted as boxplots. Each box shows the middle 50% of the data and the line inside is the median of the data. For each element, the pink box represents the mass distribution in mmNPs and the black box is for that of smNPs. Light blue line is the critical value ($L_{C,sp,i}$) in units of mass (fg) for each element.

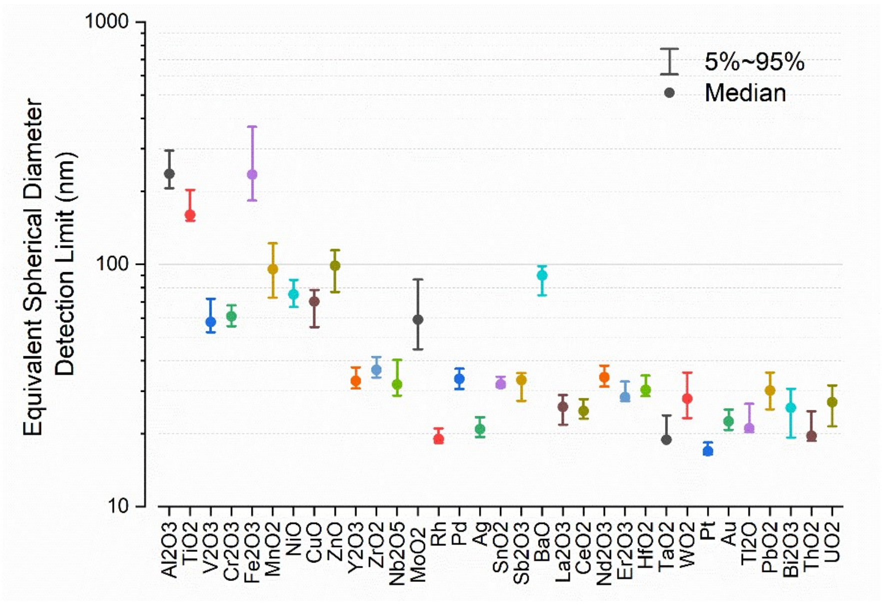

Fig. S6. Average equivalent spherical diameter detection limits of oxide forms of elements measured for the 10 analyses of the different WWTP samples (I1-I5 and E1-E5) from the Nov. sampling. For platinum-group and noble metals, no oxide forms are reported. The detection limits, which are equivalent to the critical values used to identify single-particle signals, are a function of background concentrations, element sensitivities that depend on the analyte isotope, and densities of the given oxides and metals. For example, the elevated detection limit of BaO compared to La_2O_3 comes from the fact that Ba was quantified using a minor isotope (^{137}Ba) and the barium background level is, in general, more than 10 times higher than that of lanthanum in the WWTP samples. More details on all the element sensitivities, background count rates, and critical values are provided in the SI excel spreadsheet.

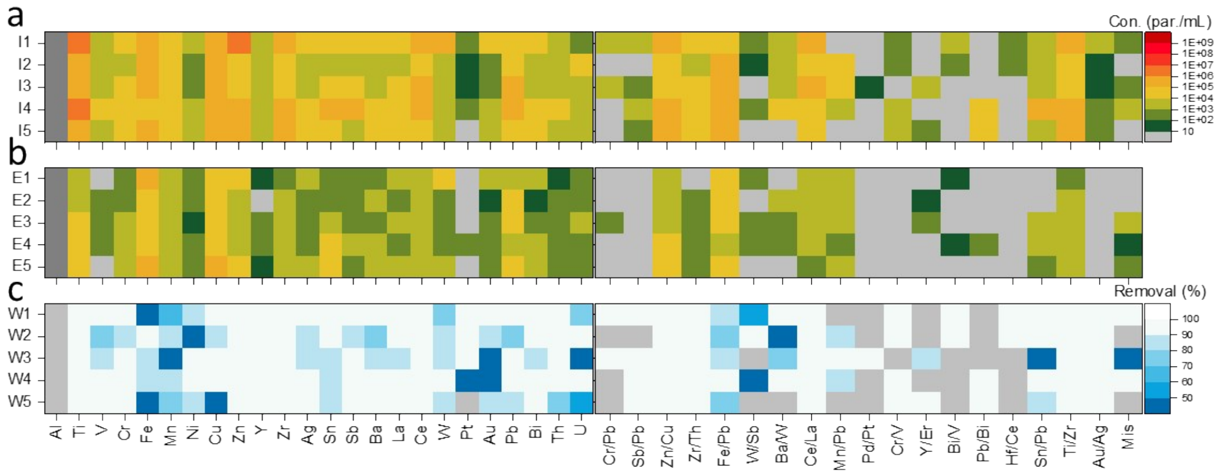

Fi

g. S7. smNPs and mmNPs detection across different WWTPs for samples collected in May 2019. Heat maps show the particle number concentrations in influent (**a**), effluent (**b**) and the PNC-based removal efficiency of each NP type in percentage (**c**). mmNP clusters are labels accorded to the two most abundant elements in each NP cluster. Classification of the mmNPs is obtained via hierarchical clustering analysis as described in text (see Fig. S8)

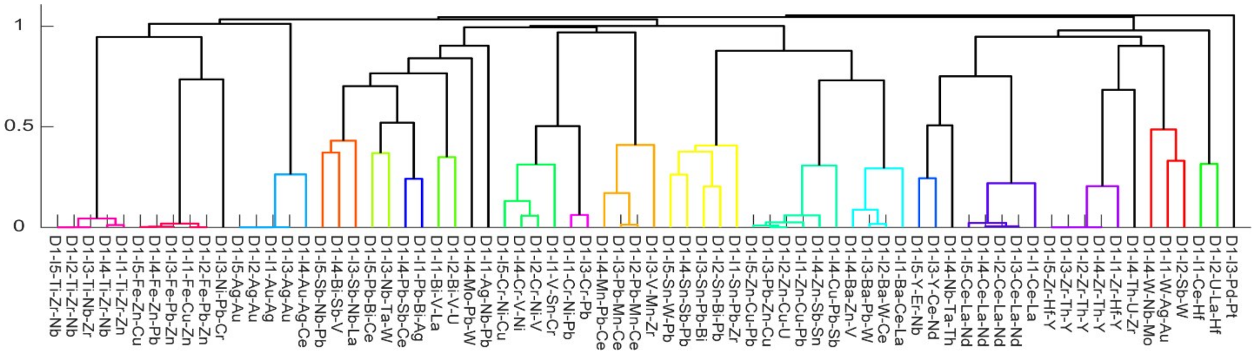

Fig. S8. Dendrogram of inter-sample hierarchical clustering analysis of I1-I5 sample collected in May 2019.

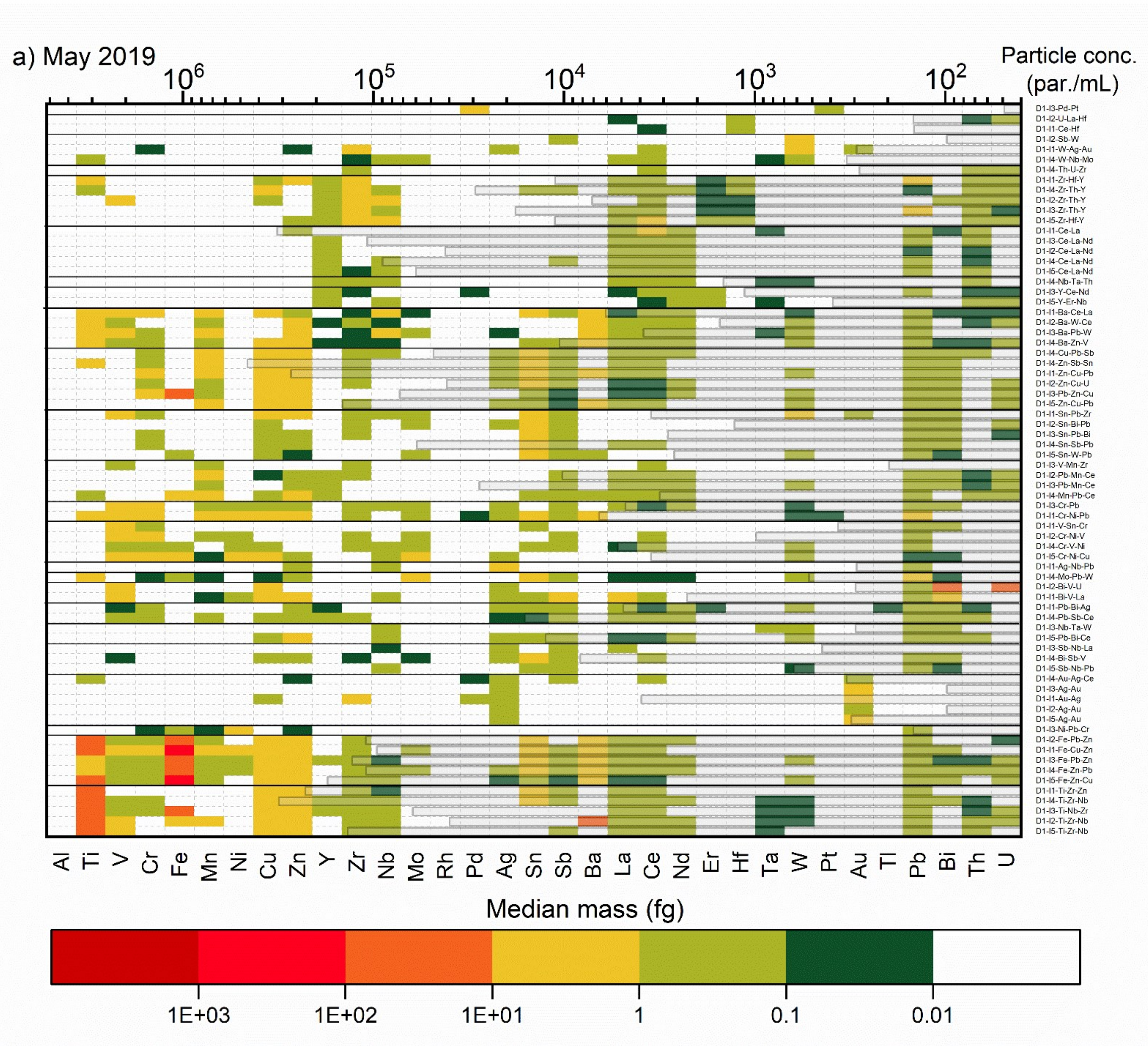

a) May 2019
Particle conc. (par./mL)
10^6
10^5
10^4
10^3
10^2
Al Ti V Cr Fe Mn Ni Cu Zn Y Zr Nb Mo Rh Pd Ag Sn Sb Ba La Ce Nd Er Hf Ta W Pt Au Tl Pb Bi Th U
Median mass (fg)
1E+03
1E+02
1E+01
1
0.1
0.01

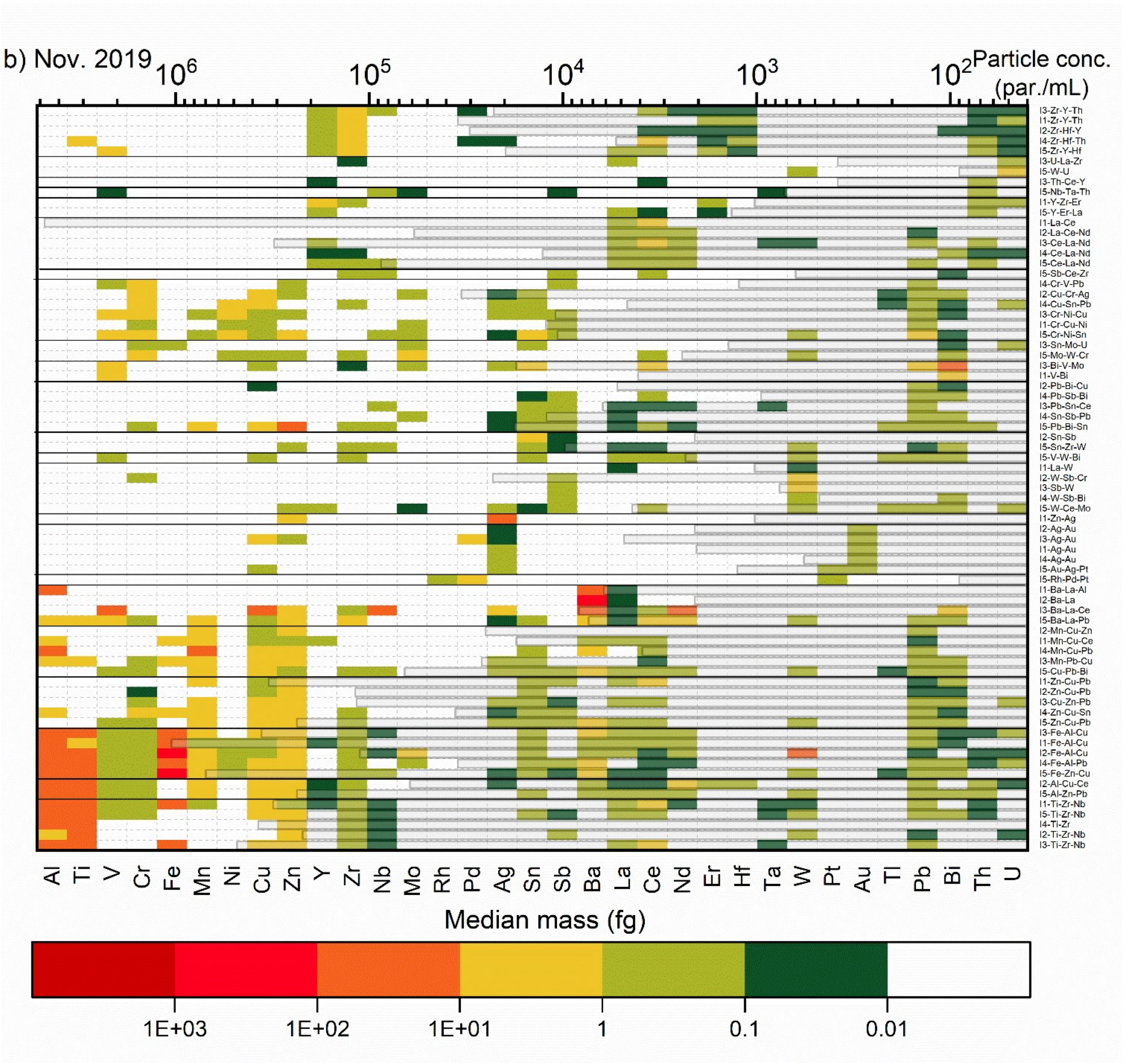

Fig. S9. Representation of all mmNP clusters in influent samples I1 to I5 in (a) May 2019 and (b) Nov 2019. Each row of the heat map shows the median mass of proxy mmNP, compose of elements exist in more than 1% of the mmNP of each cluster. Superimposed horizontal gray bars are the number concentration of each cluster. Dark lines between rows indicates the inter-sample clusters.

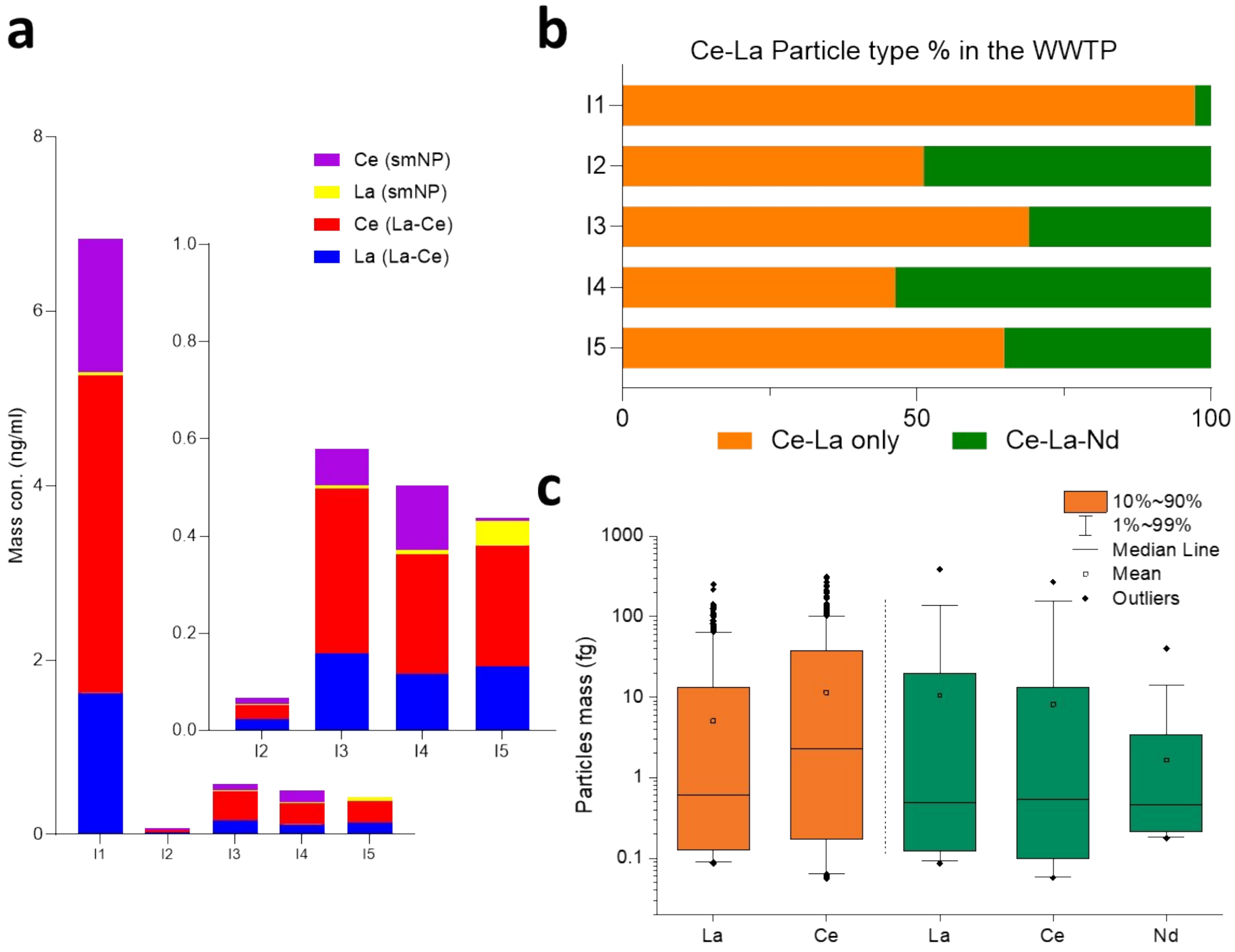

Fig. S10. Insight into the Ce-La cluster from I1-I5 collected in May 2019. **a**) Comparison of Ce and La single-metal and multi-metal total mass concentrations in I1-I5. Insert graph shows the WWTPs with substantially lower mass concentrations of Ce and La compared to that found in I1 **b**) Comparison of the particle number concentrations of Ce-La and Ce-La-Nd mmNP types. **c**) Comparison of Ce and La mass distributions in the two found sub-types of mmNPs.

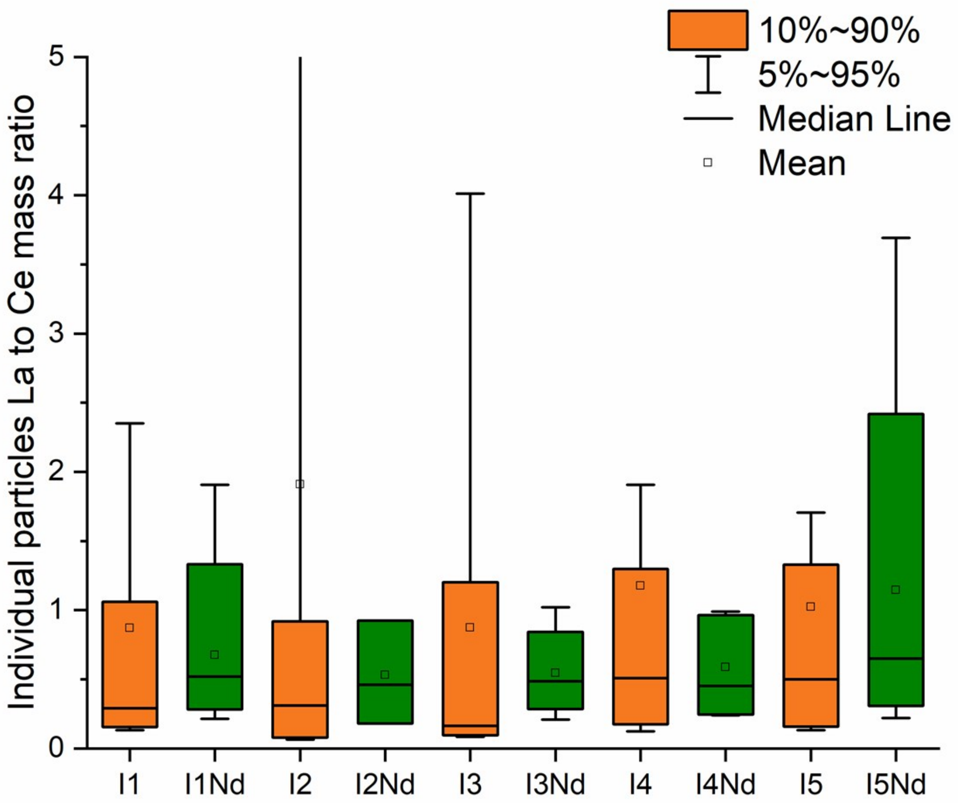

Fig. S11. Comparison of La to Ce mass ratio in the particle events detected in influent samples collected in Nov. 2019. Green boxes are the ratios for La-Ce-Nd mmNPs and the orange boxes are for La-Ce only mmNPs.

References

1. A. Gogos, J. Wielinski, A. Voegelin, F. V. Kammer and R. Kaegi, Quantification of anthropogenic and geogenic Ce in sewage sludge based on Ce oxidation state and rare earth element patterns, *Water Res X*, 2020, **9**, 100059.
2. A. Gundlach-Graham, L. Hendriks, K. Mehrabi and D. Gunther, Monte Carlo Simulation of Low-Count Signals in Time-of-Flight Mass Spectrometry and Its Application to Single-Particle Detection, *Anal. Chem.*, 2018, **90**, 11847-11855.
